# Disorder and Oxide Ion Diffusion Mechanism in La_1.54_Sr_0.46_Ga_3_O_7.27_ Melilite from Nuclear Magnetic Resonance

**DOI:** 10.1021/jacs.3c04821

**Published:** 2023-10-02

**Authors:** Lucia Corti, Dinu Iuga, John B. Claridge, Matthew J. Rosseinsky, Frédéric Blanc

**Affiliations:** † Department of Chemistry, University of Liverpool, Liverpool L69 7ZD, United Kingdom; ‡ Leverhulme Research Centre for Functional Materials Design, Materials Innovation Factory, University of Liverpool, Liverpool L69 7ZD, United Kingdom; § Department of Physics, University of Warwick, Coventry CV4 7AL, United Kingdom; ∥ Stephenson Institute for Renewable Energy, University of Liverpool, Liverpool L69 7ZF, United Kingdom

## Abstract

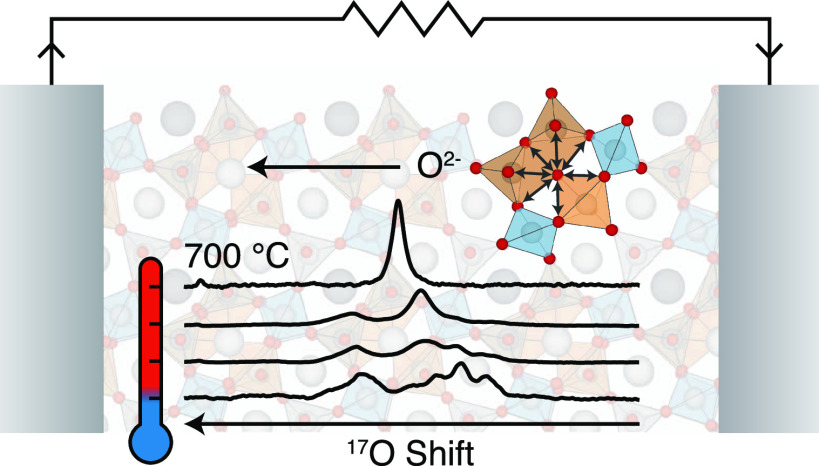

Layered tetrahedral network melilite is a promising structural family of fast ion conductors that exhibits the flexibility required to accommodate interstitial oxide anions, leading to excellent ionic transport properties at moderate temperatures. Here, we present a combined experimental and computational magic angle spinning (MAS) nuclear magnetic resonance (NMR) approach which aims at elucidating the local configurational disorder and oxide ion diffusion mechanism in a key member of this structural family possessing the La_1.54_Sr_0.46_Ga_3_O_7.27_ composition. ^17^O and ^71^Ga MAS NMR spectra display complex spectral line shapes that could be accurately predicted using a computational ensemble-based approach to model site disorder across multiple cationic and anionic sites, thereby enabling the assignment of bridging/nonbridging oxygens and the identification of distinct gallium coordination environments. The ^17^O and ^71^Ga MAS NMR spectra of La_1.54_Sr_0.46_Ga_3_O_7.27_ display additional features not observed for the parent LaSrGa_3_O_7_ phase which are attributed to interstitial oxide ions incorporated upon cation doping and stabilized by the formation of five-coordinate Ga centers conferring framework flexibility. ^17^O high-temperature (HT) MAS NMR experiments capture exchange within the bridging oxygens at 130 °C and reveal coalescence of all oxygen signals in La_1.54_Sr_0.46_Ga_3_O_7.27_ at approximately 300 °C, indicative of the participation of both interstitial and framework oxide ions in the transport process. These results further supported by the coalescence of the ^71^Ga resonances in the ^71^Ga HT MAS NMR spectra of La_1.54_Sr_0.46_Ga_3_O_7.27_ unequivocally provide evidence of the conduction mechanism in this melilite phase and highlight the potential of MAS NMR spectroscopy to enhance the understanding of ionic motion in solid electrolytes.

## Introduction

The extensive use of fossil fuels in today’s society is a matter of concern, and much research effort has been undertaken to replace fossil fuels with alternative energy sources, such as hydrogen. The deployment of clean fuels requires efficient energy conversion devices, and fuel cells enable the interconversion of chemical and electric energy. Among the various types of fuel cells, solid oxide fuel cells (SOFCs) represent one of the cutting-edge technologies that are being considered to meet the energy demand while being respectful to the environment. However, lowering the operating temperature of SOFCs to intermediate (650–800 °C) or even lower (below 650 °C) ranges is critical for the large-scale employment of this technology,^1^ and this has motivated an extensive search for novel solid electrolyte candidates that exhibit elevated oxide ion conductivity at these temperatures and can therefore be used in intermediate- and/or low-temperature SOFCs.^2^

It is well acknowledged that ion conductivity in solid electrolytes can be enhanced by incorporating aliovalent cations into the lattice, thereby forming chemical defects such as oxide ion vacancies or interstitials. A vacancy-driven ionic conduction mechanism distinguishes solid electrolytes with fluorite and perovskite structures (e.g., Zr_1–*x*_Y_*x*_O_2–0.5*x*_,^3^ Ce_1–*x*_Gd_*x*_O_2−δ_^4^ and La_1–*x*_Sr_*x*_Ga_1–*y*_Mg_*y*_O_3–0.5(*x*+*y*)_^5^) from fast oxide ion conductors with flexible structure that can accommodate interstitial oxide ions such as oxyapatite La_10-*x*_(MO_4_)_6_O_3–0.5*x*_ (where M = Si^6^ or Ge^7^), melilite La_1+*x*_M_1–*x*_Ga_3_O_7+0.5*x*_ (where M = Sr^8^ or Ca^9^), langasite La_3_Ga_5–*x*_Ge_1+*x*_O_14+0.5*x*_,^10^ and hexagonal perovskite-related Ba_7_Nb_3.9_Mo_1.1_O_20.05_^11^ structures.

LaSrGa_3_O_7_-based solid electrolytes with melilite structure have attracted significant research interest due to the remarkably high oxide ion conductivity of the La^3+^-doped phase with La_1.54_Sr_0.46_Ga_3_O_7.27_ composition that reaches values around 0.02–0.1 S cm^–1^ in the 600–800 °C temperature range combined with its stability under a hydrogen environment below 800 °C.^8^ The oxide ion conductivity measured for La_1.54_Sr_0.46_Ga_3_O_7.27_ below 600 °C is higher than that of some of the most conductive electrolyte materials such as Ge- and Si-based lanthanum apatites (e.g., La_9.5_Ge_5.5_Al_0.5_O_26_^12^ and La_9.75_Sr_0.25_(SiO_4_)_6_O_2.895_^13^) and yttrium-stabilized zirconia Zr_1–*x*_Y_*x*_O_2–0.5x_,^3^ comparable with that in La_1–*x*_Sr_*x*_Ga_1–*y*_Mg_*y*_O_3–0.5(*x*+*y*)_ (e.g., La_0.8_Sr_0.2_Ga_0.83_Mg_0.17_O_2.815_)^14^ and lower than that of Ce_1–*x*_Gd_*x*_O_2−δ_ (e.g., Ce_0.9_Gd_0.1_O_1.95_),^4^ while it exceeds that of Ba_7_Nb_3.9_Mo_1.1_O_20_ at temperatures higher than ∼450 °C.^11^ Furthermore, La_1.54_Sr_0.46_Ga_3_O_7.27_ is a pure ionic conductor, as opposed to other solid electrolytes such as Ce_1–*x*_Gd_*x*_O_2−δ_ that are mixed ionic electronic conductors.

**Figure 1:**
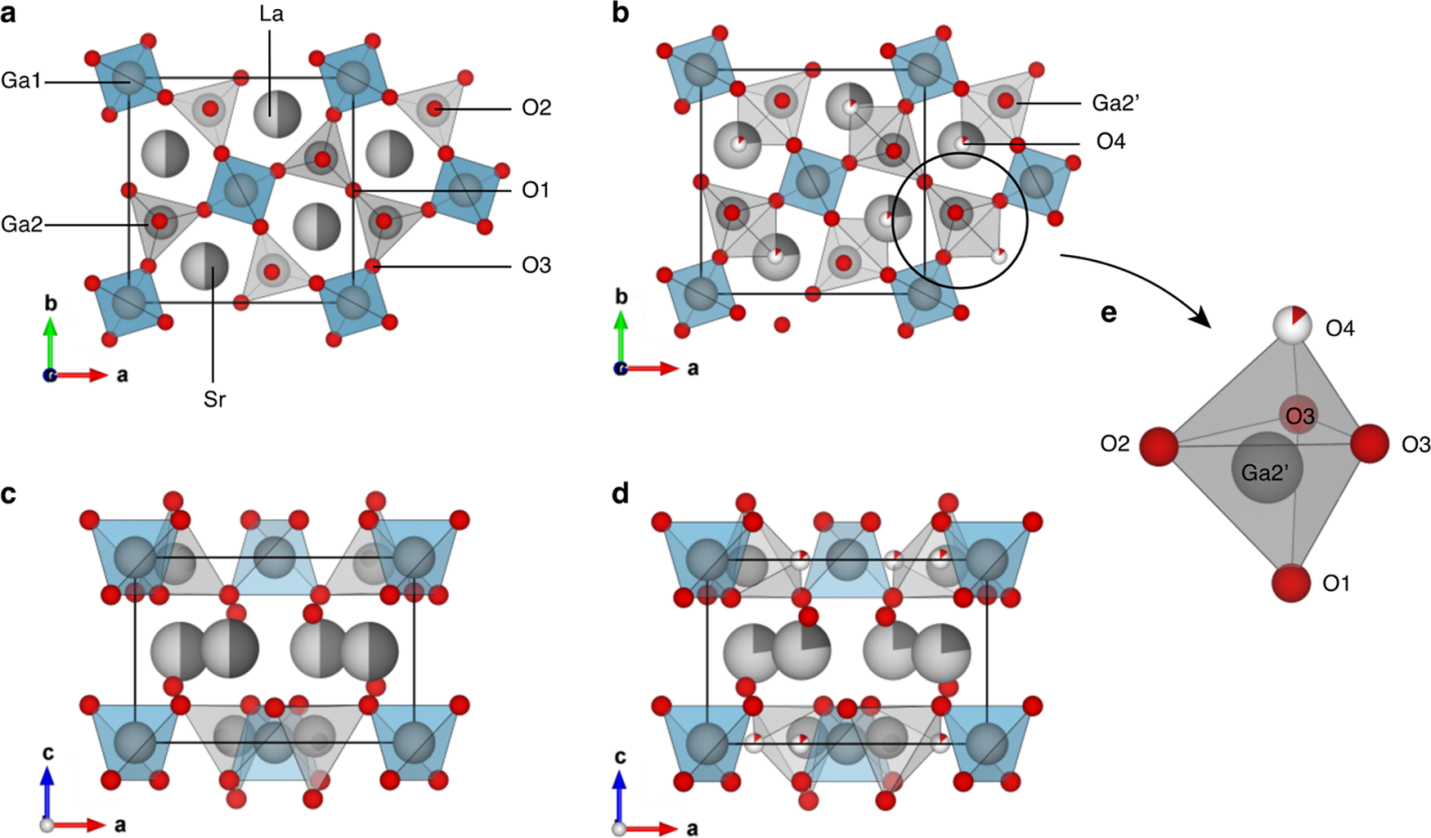
Crystal structures viewed along the (a, b) *c*-axis and the (c, d) *b*-axis of (a, c) LaSrGa_3_O_7_ and (b, d) La_1.54_Sr_0.46_Ga_3_O_7.27_.^8,16^ LaSrGa_3_O_7_ is characterized by the presence of two gallium sites (Ga1 and Ga2) and three oxygens (O1, O2, and O3) that are crystallographically distinct. (Ga1)O_4_ tetrahedra (blue) are connected to four (Ga2)O_4_ tetrahedra (gray) via O3, O1 links neighboring (Ga2)O_4_ tetrahedra and O2 is nonbridging. (e) Magnified view of the interstitial oxide ions in the La^3+^-doped La_1.54_Sr_0.46_Ga_3_O_7.27_ phase which occupy the centered position (O4) in the pentagonal rings and are accommodated in distorted trigonal bipyramidal (Ga2′)O_5_ polyhedra formed around 5-coordinate Ga sites labeled Ga2′. The average unit cells are expanded to show pentagonal rings. O, Sr, and La atoms are shown in red, dark gray, and light gray, respectively.

LaSrGa_3_O_7_ is composed of alternating layers of La^3+^/Sr^2+^ cations and anionic Ga_2_O_7_ units consisting of GaO_4_ tetrahedra connected in two dimensions via corner-sharing to construct distorted pentagonal rings (Figure 1a,c).^15^ Doping LaSrGa_3_O_7_ with La^3+^ to form La_1.54_Sr_0.46_Ga_3_O_7.27_ leads to the formation of interstitial oxide ions which are accommodated in the pentagonal rings owing to the ability of Ga^3+^ ions to change their coordination geometry and are responsible for the elevated oxide ion conductivity of the La^3+^-doped phase(Figure 1b,d,e).^8^

Investigating the structure and oxide ion diffusion mechanism in La_1+*x*_Sr_1–*x*_Ga_3_O_7+0.5*x*_ is critical to identify strategies to further enhance the transport properties of the La^3+^-doped phase and to establish design criteria for novel fast oxide ion conductors. Neutron diffraction experiments identified that the interstitial oxide ions occupy a centered position (O4) in the pentagonal ring and hinted a direct interstitial mechanism for the oxygen ion conduction.^8,16^ The position of this interstitial defect was further supported by density functional theory (DFT) and kinetic Monte Carlo (KMC) calculations which determined that the interstitial oxide ions occupy the O4 site but diffuse through an indirect interstitial mechanism.^17^ In contrast, atomistic and molecular dynamics simulations^18^ together with further neutron diffraction measurements^19^ suggested that the interstitial oxide ions occupy a slightly off-centered position labeled O5 in the pentagonal ring while also following an indirect interstitial mechanism.^18^

These earlier works on La_1.54_Sr_0.46_Ga_3_O_7.27_^8,16−19^ deploying a wide range of complementary approaches underline the potential structural complexity of this site-disordered system, in particular with regards to the interstitial ion position, and the need to further address the oxide ion conduction mechanism. This is especially important given the excellent performance of this family of SOFC electrolytes arising from the incorporation of mobile interstitial oxide ions into the framework, motivating further exploration of La_1.54_Sr_0.46_Ga_3_O_7.27_ using magic angle spinning (MAS) nuclear magnetic resonance (NMR) spectroscopy experiments, which are yet to be exploited effectively on this phase.^8,16−19^ Owing to the sensitivity of NMR to the local environment of the interacting nuclear spins, ^17^O (spin quantum number *I* = 5/2) MAS NMR is playing an important role in both the exploration of the local structure around the oxygen sites (the key element in solid oxide ion conductors) and the determination of the oxide ion dynamics across multiple time scales.^20−24^ Although the acquisition of ^17^O solid-state NMR spectra is challenged by the low natural abundance (0.037%) of the only NMR-active isotope of oxygen, ^17^O, fast oxide ion conductors can intrinsically be readily enriched in ^17^O via postsynthetic exchange with ^17^O enriched O_2_ gas.^25−27^ Furthermore, the sensitivity of the ^71^Ga (*I* = 3/2) chemical shielding and quadrupolar interactions to changes in the Ga coordination environment makes ^71^Ga NMR spectroscopy a powerful approach to access the local chemical environment and defect chemistry^28−32^ that strongly complements the average, long-range atomic structure refined from diffraction-based methods in LaSrGa_3_O_7_-based solid electrolytes. Other NMR-active nuclei in the melilite samples with the potential to reveal valuable structural information are ^139^La (*I* = 7/2) and ^87^Sr (*I* = 9/2).

The aim of this work is to exploit the high-field and high-temperature capabilities of MAS NMR spectroscopy to (i) gain insight into the configurational disorder in La_1+*x*_Sr_1–*x*_Ga_3_O_7+0.5*x*_ with *x* = 0 and 0.54 which arises from a broad array of possible distributions of the La^3+^/Sr^2+^ cations and interstitial defects within the lattice and (ii) unequivocally elucidate the oxide ion diffusion mechanism in the La^3+^-doped phase. In particular, detailed information on the local atomic environment around the O and Ga sites is obtained from ^17^O and ^71^Ga MAS NMR spectroscopy, augmented by the computation of the NMR parameters using the gauge including projector augmented waves (GIPAW) method^33^ on a symmetry-adapted configurational ensemble obtained with a site occupancy disorder (SOD) approach,^34^ while high-temperature ^17^O MAS NMR up to 700 °C at very high field (20 T) is employed to access the motion of the oxide ions and unravel the conduction mechanism. The SOD approach has already been employed in conjunction with experimental NMR spectroscopy in pioneering work^35^ that focuses on the Sn^4+^/Ti^4+^ cation distribution in the 6-coordinate B site of the A_2_B_2_O_7_ pyrochlore structure used as a case study, thereby showcasing the great potential of this method for the understanding of configurational disorder. Here, we capitalize on this recently published work and expand the approach to the significantly more complex melilite phases which require the modeling of correlated disorder across multiple cationic and anionic sites that originates as O^2–^ interstitial defects are introduced in the melilite lattice by substituting Sr^2+^ cations with La^3+^ cations.

## Experimental Section

### Materials Synthesis

The synthesis of LaSrGa_3_O_7_ and La_1.54_Sr_0.46_Ga_3_O_7.27_ was carried out as described in previous work.^8^ To enable the collection of ^17^O solid-state NMR data, LaSrGa_3_O_7_ and La_1.54_Sr_0.46_Ga_3_O_7.27_ were ^17^O enriched using a standard annealing procedure^25^ based on postsynthetic exchange with 60% ^17^O enriched O_2_ gas (Isotec). In a typical experiment, 60% ^17^O enriched O_2_ gas (0.58 mmol) was added to a quartz tube containing a melilite composition (1 mmol) precooled at −196 °C in liquid nitrogen. The samples were then brought back to room temperature and heated to 750 °C at a rate of 5 K min^–1^, kept at 750 °C for 24 h, and then cooled back to room temperature at a rate of 5 K min^–1^. Based on mass balance analysis of the ^17^O enriched O_2_ gas and melilite sample used in the enrichment procedure and assuming a statistical distribution of the O isotopes, the ^17^O content in the samples is expected to be approximately 8.5%. Powder X-ray diffraction (PXRD) measurements were performed on a Panalytical X’pert Pro Multi-Purpose X-ray diffractometer with Co Kα_1_ radiation (λ = 1.78901 Å).

### Solid-State MAS NMR Experiments

#### ^17^O Solid-State MAS NMR Experiments at Room Temperature

^17^O NMR spectra at room temperature were recorded on a 9.4 T Bruker Avance III HD spectrometer, an 18.8 T Bruker Avance Neo spectrometer and a 20 T Bruker Avance Neo spectrometer, respectively, equipped with a 4 mm triple resonance HXY probe (in double-resonance mode) tuned to X = ^17^O at the Larmor frequency ν_0_ = 54.25 MHz under a MAS rate of ν_r_ = 10.0 kHz, a 1.3 mm double-resonance HX probe (unless otherwise specified) tuned to X = ^17^O at ν_0_ = 108.50 MHz under a MAS rate of ν_r_ = 60.0 kHz and a 4 mm HX high-temperature MAS probe tuned to ν_0_ = 115.28 MHz under a MAS rate of ν_r_ = 10.0 kHz. One pulse spectra were recorded using an experimentally optimized 30° flip angle pulse with radio frequency (rf) pulse amplitude of ν_1_ = 50 kHz at 9.4 T, ν_1_ = 100 kHz at 18.8 T and ν_1_ = 42 kHz at 20 T, and a recycle delay of 5 × *T*_1_, where *T*_1_ is the spin–lattice relaxation time constant determined from saturation recovery experiments (fitted as discussed below). Short flip angle pulses were used to minimize effects arising from differences in the nutation frequency of quadrupolar nuclei, thereby obtaining quantitative spectra when combined with suitable recycle delays that allow complete equilibration of the nuclear spin system.^36^ Two-dimensional *z*-filter^37^ triple-quantum magic angle spinning (3QMAS) experiments^38^ were performed at 9.4 and 18.8 T. A 3.2 mm HX probe spinning at ν_r_ = 20.0 kHz was employed to record 3QMAS experiments at 18.8 T to enhance the signal-to-noise ratio as opposed to the 1.3 mm HX probe used to record one-dimensional spectra. 3QMAS experiments were acquired using excitation and reconversion pulses with rf field amplitude of 58.5 kHz at 9.4 T and 47.6 kHz at 18.8 T and π/2 selective pulses with rf field amplitude of 15 kHz at 9.4 T and either 20 kHz (for LaSrGa_3_^17^O_7_) or 30 kHz (for La_1.54_Sr_0.46_Ga_3_^17^O_7.27_) at 18.8 T. The isotropic chemical shifts δ_iso,cs_ and quadrupole products *P*_Q_ were determined from the position of the center of gravity of the resonances projected along the isotropic (δ_*f*_1__) and anisotropic (δ_*f*_2__) dimensions of the sheared 3QMAS spectra using eqs 1 and 2(39)
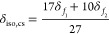
1and, for spin *I* = 5/2 nuclei,
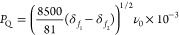
2

The ^17^O quadrupolar coupling constants *C*_Q_ and quadrupolar asymmetry parameters η_Q_ were determined by fitting the cross sections parallel to the δ_*f*_2__ dimension while using eq 3
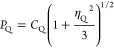
3

The NMR shift δ (i.e., the center of gravity of the signals) is the sum of the isotropic chemical shift δ_iso,cs_ and the quadrupolar induced shift δ_QIS_ which for spin *I* = 5/2 nuclei can be determined from eq 4.
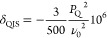
4

The magic angle was adjusted by using the ^79^Br NMR signal of KBr. All ^17^O NMR experiments were carried out on ^17^O enriched samples and are reported relative to the ^17^O signal of H_2_O at 0 ppm. The NMR data were processed and simulated using TopSpin 4.0.9.

#### ^17^O Variable-Temperature MAS NMR Experiments

^17^O NMR high-temperature spectra in the 20–700 °C temperature range were acquired on a 20 T Bruker Avance Neo spectrometer equipped with a 7 mm laser-heated single-resonance X MAS probe^40^ under a MAS rate ν_r_ = 4.0 kHz. Data in the 20–300 °C temperature range were additionally recorded with a 4 mm high-temperature double-resonance HX MAS probe under a MAS rate of ν_r_ = 10.0 kHz owing to the higher MAS rates attainable with this probe. High-temperature ^17^O one pulse experiments were acquired using a recycle delay of at least 5 × *T*_1_ and an experimentally optimized 30° flip angle pulse with rf field amplitude of either 20.0 kHz (7 mm probe) or 41.8 kHz (4 mm probe), with the exceptions of data obtained for LaSrGa_3_^17^O_7_ with the 7 mm laser-heated probe which were acquired with a 90° flip angle pulse and a recycle delay of at least 1.3 × *T*_1_ due to the long *T*_1_ relaxation time constants. ^17^O–^17^O 2D exchange spectroscopy (EXSY) spectra of La_1.54_Sr_0.46_Ga_3_^17^O_7.27_ were recorded with the 4 mm high-temperature probe between 20 and 150 °C using mixing times up to 100 ms, a recycle delay of 1.3 × *T*_1_ and at least 90 *t*_1_ increments.

Saturation recovery experiments were recorded between 20 and 700 °C with either the 7 mm laser probe or the 4 mm high-temperature probe to extract spin–lattice relaxation rates in the laboratory frame *T*_1_^–1^ as a function of reciprocal temperature *T*. A saturation block consisting of a train of 90° flip angle pulses with an rf field amplitude of either 20 kHz (7 mm probe) or 41.8 kHz (4 mm probe) separated by short delays was used to saturate the spins. The duration of those delays δ (between 60 μs and 1.05 ms) and the number of pulses (between 10 and 100) in the saturation block were set based on the *T*_1_ value at each temperature ensuring that saturation of both central and satellite transitions was achieved and taking into consideration the probe safety. Data were fitted to the stretch exponential function in eq 5 to account for the *T*_1_ distribution arising from the presence of several partially unresolved ^17^O signals as well as the temperature gradient across the rotor
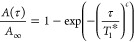
5where *A*(τ) and *A*_∞_ are the areas of the overlapping ^17^O signals at time τ and infinity, respectively; *T*_1_^*^ is the characteristic time constant, and *c* is the stretch exponent which reflects the distribution of *T*_1_^*^ values and was measured to vary from ∼0.56 to 1, the upper limit corresponding the monoexponential function. *T*_1_^*^ and *c* are phenomenological parameters that parametrize the underlying distribution of relaxation times and have no direct physical meaning. The mean *T*_1_ value can be expressed in terms of *T*_1_^*^ and *c* without explicit knowledge of the shape of the relaxation time distribution function using eq 6(41)
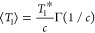
6where Γ indicates the γ function.^42^Equation 6 simplifies to ⟨*T*_1_⟩ = *T*_1_^*^ for monoexponential functions (i.e., *c* = 1). Weights determined from kernel density estimation were included in the *T*_1_ fitting procedure because data were not sampled at uniform intervals.

The temperature calibration was performed using the ^207^Pb chemical shift thermometer of Pb(NO_3_)_2_^43^ for the 4 mm high-temperature HX MAS probe and the ^79^Br chemical shift thermometer of KBr,^44^ supported by the identification of changes in the ^23^Na spectral line shapes of Na_3_AlF_6_ across the phase transition from monoclinic to orthorhombic at ∼550 °C^45^ for the 7 mm laser-heated X MAS probe. These calibrations revealed temperature differences across the rotor up to ∼50 °C at 700 °C for the 7 mm probe and ∼5 °C at 200 °C for the 4 mm probe. All of the temperatures reported correspond to the actual sample temperatures.

#### ^71^Ga MAS NMR Experiments

All ^71^Ga NMR experiments at room temperature were carried out on an 18.8 T Bruker Avance Neo spectrometer equipped with a 1.3 mm double-resonance HX probe tuned to X = ^71^Ga at ν_0_ = 244.01 MHz under a MAS rate of ν_r_ = 60.0 kHz. Rotor-synchronized Hahn echo experiments were performed using experimentally optimized π/2 - π pulses at a rf field amplitude of 200 kHz and a recycle delay of 5 × *T*_1_, where *T*_1_ was determined from ^71^Ga saturation recovery experiments performed with a saturation block consisting of a train of 100 pulses at a rf field amplitude of 200 kHz separated by delays of 1 ms. Two-dimensional *z*-filter 3QMAS spectra were acquired using excitation and reconversion pulses with an rf field amplitude of 200 kHz and π/2 selective pulses with an rf field amplitude of 20 kHz. The δ_iso,cs_ and *P*_Q_ values were extracted using eq 1 and 7, with the latter being valid for spin *I* = 3/2 nuclei.
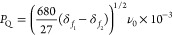
7

Equation 8, valid for spin *I* = 3/2 nuclei, was used to determine the δ_QIS_ values.
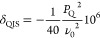
8

^71^Ga spectra are referenced to a 1 M solution of Ga(NO_3_)_3_ in H_2_O at 0 ppm.

#### ^71^Ga Variable-Temperature MAS NMR Experiments

^71^Ga NMR high-temperature spectra between 20 and 700 °C were recorded on a 20 T Bruker Avance Neo spectrometer equipped with a 7 mm laser-heated single-resonance X MAS probe^40^ tuned to X = ^71^Ga at ν_0_ = 259.34 MHz under a MAS rate ν_r_ = 4.0 kHz. The spectra were acquired using the one pulse sequence, a recycle delay of approximately 5 × *T*_1_ and experimentally optimized 90° flip angle pulses with rf field amplitude of 24.5 kHz.

### Computations

The SOD approach^34^ was employed to generate a set of 2 and 18 symmetrically inequivalent configurations (i.e., not related by an isometric transformation), respectively, for a LaSrGa_3_O_7_ unit cell and a La_1.5_Sr_0.5_Ga_3_O_7.25_ 1 × 1 × 2 super cell (based on the unit cell parameters revealed by the crystallographic data of the site occupancy disordered La^3+^-doped phase).^16^ A 1 × 1 × 2 super cell with La_1.5_Sr_0.5_Ga_3_O_7.25_ composition was chosen as it resembles the experimental composition La_1.54_Sr_0.46_Ga_3_O_7.27_, while maintaining a low computational cost. The structural data with interstitial oxide ions occupying the centered O4 sites were used to obtain the symmetrically inequivalent configurations for the La^3+^-doped phase.^8,16^ All calculations were performed using the CASTEP (version 20.11) package.^46^ The geometry of each configuration was optimized using plane-wave DFT^47^ with periodic boundary conditions. All atomic coordinates and unit cell parameters were optimized using on-the-fly generated ultrasoft pseudopotentials,^48^ the zeroth-order regular approximation (ZORA) approach^49^ to treat scalar relativistic effects and the Perdew–Burke–Ernzerhof (PBE)^50^ exchange-correlation functional. The plane-wave cutoff energy was set to 800 eV and the Brillouin zone was sampled using a 2 × 2 × 3 and a 2 × 2 × 2 Monkhorst–Pack^51^ k-point grid for LaSrGa_3_O_7_ and La_1.5_Sr_0.5_Ga_3_O_7.25_, respectively. Further increasing the accuracy of the cutoff energy and *k*-point density led to changes in energy equal to or smaller than 3.2 meV/atom. The electronic energy was optimized self-consistently until a threshold of 1 × 10^–9^ eV/atom was reached as suggested previously.^35^ In the geometry optimization, the convergence thresholds for the maximum energy change, maximum force, maximum stress, and maximum displacement were, respectively, set to 1 × 10^–5^ eV/atom, 5 × 10^–2^ eV/Å, 1 × 10^–1^ GPa, and 1 × 10^–3^ Å. NMR parameters were calculated on the optimized geometries using the GIPAW approach^33,52^ and applying the same parameters as in the geometry optimization. The calculations yield the absolute shielding tensor, **σ**, in the crystal frame. Diagonalization of the symmetric part of the absolute shielding tensor gives the three principal components (σ_*xx*_, σ_*yy*_, σ_*zz*_) ordered according to the Haeberlen convention^53^ such that |σ_*zz*_ – σ_iso_| ≥ |σ_*xx*_ – σ_iso_| ≥ |σ_*yy*_ – σ_iso_|. The absolute chemical shielding tensor is expressed in terms of the isotropic chemical shielding σ_iso,cs_ = 1/3 (σ_*xx*_ + σ_*yy*_ + σ_*zz*_), the anisotropic chemical shielding σ_aniso,cs_ = σ_*zz*_ + 1/2 (σ_*xx*_ + σ_*yy*_) and the asymmetry parameter η = (σ_*yy*_ – σ_*xx*_)/(σ_*zz*_ – σ_iso_). To enable the comparison between computational and experimental results, the computed σ_iso,cs_ and σ_aniso,cs_ terms were converted into isotropic chemical shift δ_iso,cs_ and anisotropic chemical shift δ_aniso,cs_ using δ_iso,cs_ = σ_ref_ + *m*σ_iso,cs_ and δ_aniso,cs_ = *mσ*_aniso,cs_ with σ_ref_ = 222.02 ppm and *m* = −0.872 for ^17^O and σ_ref_ = 1442.22 ppm and *m* = −0.821 for ^71^Ga following a known procedure^54^ which also aims at minimizing the systematic errors present in the calculations. The calculations generate the traceless electric field gradient tensor ***V*** with its three principal components (*V*_*xx*_, *V*_*yy*_, *V*_*zz*_) ordered such that |*V*_*zz*_| ≥ |*V*_*yy*_| ≥ |*V*_*xx*_|. The electric field gradient tensor can be expressed in terms of the quadrupolar coupling constant *C*_Q_ = *eQV*_*zz*_/*h* and the quadrupolar asymmetry parameter η_Q_ = (*V*_*xx*_ – *V*_*yy*_)/*V*_*zz*_.

### Numerical Simulations

The SIMPSON package^55^ was used to simulate the ^17^O and ^71^Ga NMR spectra from the NMR parameters computed with the GIPAW approach (δ_iso,cs_, the reduced anisotropic chemical shift δ_aniso,red,cs_ = δ_*zz*_ – δ_iso,cs_, η, *C*_Q_, and η_Q_) taking into account both the electric field and chemical shift anisotropy tensors. While both CASTEP and SIMPSON adopt the Haeberlen convention to describe the chemical shift tensor, in SIMPSON the anisotropic contribution to the chemical shift tensor is expressed in terms of the reduced anisotropic chemical shift. The spectra were simulated with the gcompute method using the rep2000 crystal file for powder averaging.^56^^17^O and ^71^Ga NMR spectra were simulated for each configuration as the sum of the contributions from each oxygen or gallium site. The NMR spectra simulated for each configuration were weighted by the corresponding configurational degeneracy and subsequently summed to obtain the total simulated NMR spectra in the high-temperature limit, *e*^–Δ*E*/*k*_B_*T*^ → 1. In some instances, the relative energy of each configuration was also taken into account.

## Results and Discussion

### Configurational Disorder

To enable the detection of ^17^O NMR signals, the LaSrGa_3_O_7_ and La_1.54_Sr_0.46_Ga_3_O_7.27_ samples were postsynthetically ^17^O enriched using an annealing procedure under an atmosphere of ^17^O enriched O_2_ gas at 750 °C, which is below the temperature of partial decomposition.^57^ While several other methods for ^17^O enrichment have been successfully developed,^58−61^ annealing is the most robust approach for these melilites owing to their fast oxide ion mobility. PXRD patterns (Figures S1a,b and S2a,b in the Supporting Information) of the natural abundance and ^17^O enriched melilites confirm that the ^17^O enrichment procedure does not alter the long-range structure and composition of the samples while revealing the presence of very small amounts of La(Sr)GaO_3_ perovskite, present in the pristine samples and also detected in both the ^17^O and ^71^Ga MAS NMR spectra of LaSrGa_3_O_7_ and La_1.54_Sr_0.46_Ga_3_O_7.27_, as discussed below. The intensity of the La(Sr)GaO_3_ signal observed at a shift δ of ∼56 ppm^31^ in the ^71^Ga Hahn echo MAS NMR spectra of the natural abundance and ^17^O enriched melilite phases (Figure S3) confirms that the enrichment strategy does not lead to an increase in the amount of La(Sr)GaO_3_ present in the samples. The ^17^O content in the ^17^O enriched melilite samples estimated using the ZrO_2_ rotor as an internal standard for oxygen at natural abundance is approximately 8%, in agreement with the ^17^O enrichment level of 8.5% expected on the basis of mass balance analysis of the ^17^O enriched O_2_ gas and melilite sample used in the enrichment procedure. As discussed in more detail below, homogeneous ^17^O enrichment is importantly attained for La_1.54_Sr_0.46_Ga_3_O_7.27_, while the LaSrGa_3_^17^O_7_ samples are nonhomogeneously enriched in ^17^O, reflecting the less efficient ionic diffusion observed for this phase.

**Figure 2:**
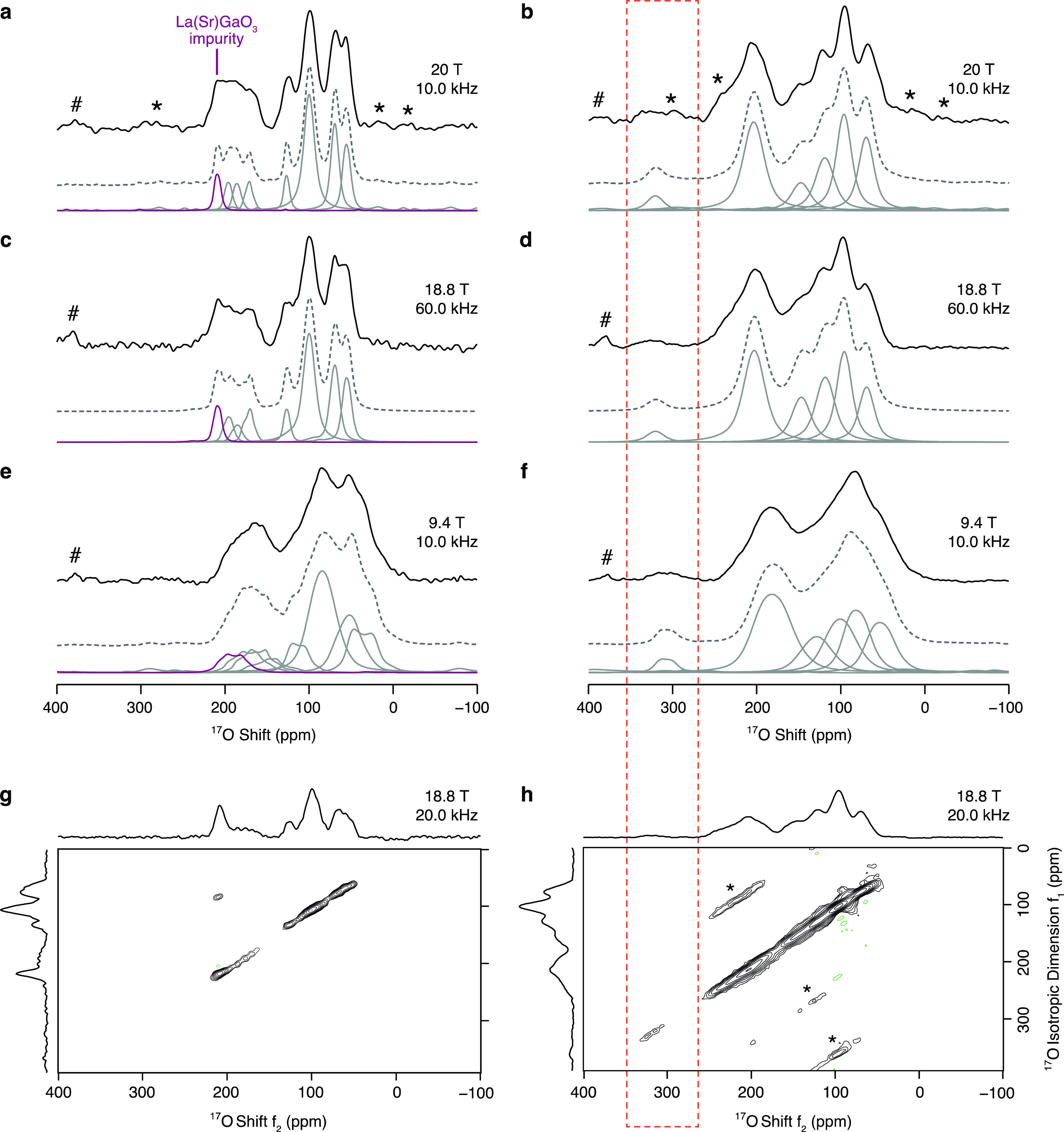
^17^O MAS NMR spectra recorded at (a, b) 20 T with MAS rate ν_r_ = 10.0 kHz, (c, d) 18.8 T with a MAS rate ν_r_ = 60.0 kHz, and (e, f) 9.4 T with a MAS rate ν_r_ = 10.0 kHz of (a, c, e) LaSrGa_3_^17^O_7_ and (b, d, f) La_1.54_Sr_0.46_Ga_3_^17^O_7.27_. The simulated spectra (dashed gray lines) and deconvoluted signals (solid gray lines) are shown below the experimental data (solid black lines) at each field. The signal at δ_iso,cs_ = 215 ppm (solid purple lines) is assigned to a La(Sr)GaO_3_ impurity.^31^ The hash symbol (#) indicates the signal of the ZrO_2_ rotor, and the asterisks (*) denote the spinning sidebands. ^17^O 3QMAS spectra of (g) LaSrGa_3_^17^O_7_ and (h) La_1.54_Sr_0.46_Ga_3_^17^O_7.27_ recorded at 18.8 T with a MAS rate ν_r_ = 20.0 kHz displaying projections of the 2D 3QMAS spectra along the *f*_1_ (left) and *f*_2_ (top) dimensions. The dashed orange box surrounds the signal assigned to interstitial defects O4.

Room temperature ^17^O MAS NMR spectra of LaSrGa_3_^17^O_7_ and La_1.54_Sr_0.46_Ga_3_^17^O_7.27_ were recorded at several external magnetic field strengths from 9.4 to 20 T (Figure 2a–f) to investigate the configurational disorder and observe the interstitial oxygen sites. The employment of high-field NMR spectroscopy is particularly beneficial for the acquisition of ^17^O MAS NMR spectra owing to the quadrupolar nature of the only NMR-active isotope of oxygen, ^17^O, which results in a fourth-rank second-order quadrupolar broadening of the NMR signals that is not averaged to zero even under rapid sample spinning at the magic angle, but which can be substantially reduced by utilizing high external magnetic field strengths, as observed in Figure 2a–f, to resolve signals corresponding to oxide ions in slightly different chemical environments. Nevertheless, the ^17^O MAS NMR spectra of LaSrGa_3_^17^O_7_ display several partially overlapping signals in the 0–250 ppm region of chemical shifts, even when performing the experiments at high external magnetic field strength (up to 20 T) or under fast MAS rates (up to 60.0 kHz). The signals appear broad, owing to the aforementioned second-order quadrupolar broadening superimposed on an inhomogeneous broadening arising from a distribution of chemical shifts typical of disordered systems. A common approach to remove this fourth-rank anisotropic term and obtain high-resolution spectra of half-integer quadrupolar nuclei is to record 2D 3QMAS spectra. The corresponding spectrum of LaSrGa_3_^17^O_7_ (Figure 2g) displays four distinct signals in the 0–150 ppm region, at least three additional signals at slightly higher chemical shifts between 150 and 200 ppm and a sharp signal at a shift δ_iso,cs_ ∼ 215 ppm that is assigned to the La(Sr)GaO_3_ impurity (note that the high intensity of this resonance likely arises from the oxide ion transport properties of this phase which are more efficient than those of the undoped melilite).^8^ The projections of the signals in the 3QMAS spectrum along the isotropic *f*_1_ and anisotropic *f*_2_ dimensions enable the extraction of the ^17^O quadrupolar NMR parameters (*i.e., δ*_iso,cs_, *C*_Q_, and η_Q_), as described in the Experimental Section. The ^17^O NMR parameters derived from the 3QMAS spectrum are listed in Table 1 and accurately reproduce the ^17^O MAS NMR spectra of LaSrGa_3_^17^O_7_ at all fields. ^17^O chemical shift anisotropy interactions are not included in the fitting procedure because of their negligible contribution to the overall spectrum, based on previous work^54,62^ and as discussed in more detail in the Supporting Information (Figure S4a–f).

**Table 1:** NMR Parameters Obtained from the 1D MAS and 2D 3QMAS NMR Data of LaSrGa_3_O_7_ and La_1.54_Sr_0.46_Ga_3_O_7.27_, Highlighting the Spectral Assignment Deduced from the Computed NMR Parameters

composition	δ_iso,cs_ (ppm)^a^	*C*_Q_ (MHz)^b^,^c^	η_Q_^b^	Assignment
LaSrGa_3_O_7_	61(4)	3.5(2)	0.1(2)	O3
	74(4)	3.1(2)	0.6(2)	O3
	105(6)	3.1(2)	0.5(2)	O3
	131(4)	3.0(1)	0.2(2)	O1
	179(3)	3.7(3)	0.7(2)	O2
	193(6)	3.8(5)	0.5(3)	O2
	203(7)	3.8(4)	0.5(3)	O2
	215(4)	3.5(2)	0.5(2)	La(Sr)Ga**O**_3_
	∼190^d^	–d	–d	Ga2
	225–228	4.2–7.2^c^	–	Ga1
La_1.54_Sr_0.46_Ga_3_O_7.27_	75(2)	3.2(5)	0.4(2)	O3
	101(5)	3.1(3)	0.3(2)	O3
	124(2)	3.4(2)	0.5(2)	O1, O3
	153(5)	3.3(2)	0.6(3)	O1, O3
	210(9)	3.7(2)	0.4(3)	O2
	325(9)	2.9(3)	0.2(3)	O4
	∼90^d^	–d	–d	Ga2′
	∼140^d^	–d	–d	Ga2
	211–220	4.0–9.3^c^	–	Ga1

a Experimental isotropic shift obtained from the projections along the isotropic δ_*f*_1__ and anisotropic δ_*f*_2__ dimensions of the signals in the sheared 3QMAS spectra.

b Values evaluated by fitting the cross sections of the signals in the ^17^O 3QMAS spectra parallel to the δ_*f*_2__ dimension and keeping the experimentally determined *P*_Q_ value fixed. See the Experimental Section for further details.

c *P*_Q_ values for ^71^Ga determined from the sheared 3QMAS spectra using eq 7.

d Only an estimation of the ^71^Ga shift δ from the ^71^Ga Hahn echo MAS NMR spectra is provided because Ga2 and Ga2′ are not observed in the ^71^Ga 3QMAS spectrum under the experimental conditions used.

Importantly, the ^17^O MAS NMR spectra of ^17^O enriched La_1.54_Sr_0.46_Ga_3_^17^O_7.27_ (Figure 2b,d,f) reveal the presence of a strongly deshielded signal at δ ∼ 320 ppm which is not observed in the spectra of LaSrGa_3_^17^O_7_ and which is assigned to the interstitial oxide ions. Similar deshielded signals were observed in the ^17^O MAS NMR spectra of La_3_Ga_5–*x*_Ge_1+*x*_O_14+0.5*x*_,^10^ La_8_Y_2_Ge_6_O_27_^63^ and La_8_CaYGe_6_O_26.5_,^63^ where the signal assigned to interstitial oxide ions accommodated in a five-coordinate Ga/Ge unit appeared more deshielded than the signal corresponding to oxide ions in Ga/Ge tetrahedra. The remaining resonances observed in the 0–280 ppm region of the spectra significantly overlap and are generally broader than those observed for LaSrGa_3_^17^O_7_, likely owing to the presence of second-order quadrupolar effects combined with an intensified inhomogeneous distribution of chemical shifts suggesting enhanced structural disorder in the La^3+^-doped phase. Comparison of the shape of the signals in the LaSrGa_3_^17^O_7_ and La_1.54_Sr_0.46_Ga_3_^17^O_7.27_ 3QMAS spectra at 9.4 T (Figure S5a,b) confirms enhanced structural disorder in the La-doped phase, as the La_1.54_Sr_0.46_Ga_3_^17^O_7.27_ signals are significantly elongated along the diagonal as opposed to the LaSrGa_3_^17^O_7_ signals which are distributed parallel to the anisotropic *f*_2_ dimension. This effect is less visible in the data at 18.8 T (Figure 2g,h) owing to the reduced quadrupolar effects at higher magnetic field strengths. The ^17^O 3QMAS spectrum of La_1.54_Sr_0.46_Ga_3_^17^O_7.27_ at 18.8 T clearly shows four distinct signals at δ_*f*_2__ < 170 ppm, at least one signal but possibly more in the 170 ppm < δ_*f*_2__ < 250 ppm region and one signal at δ_*f*_2__ of ∼320 ppm. The ^17^O NMR parameters extracted from the 3QMAS spectrum (Figure 2h) are listed in Table 1 and accurately reproduce the ^17^O MAS NMR spectra of La_1.54_Sr_0.46_Ga_3_^17^O_7.27_ at all fields, too. The projection of the ^17^O 3QMAS spectrum along the *f*_2_ dimension matches the line shape of the corresponding ^17^O MAS NMR spectrum, indicating efficient excitation of all ^17^O resonances in the ^17^O 3QMAS experiment.

The interpretation of the ^17^O MAS NMR spectra solely based on chemical shielding arguments is challenged by the complexity of the observed spectral line shape. Computationally assisted assignment of NMR signals represents a powerful approach to guide the interpretation of NMR spectra and is often based on the computation of the NMR parameters using the GIPAW method^33,52^ for periodic solids. Nevertheless, the computational modeling of site-disordered solids is made particularly difficult by the local configurational complexity arising from fractional site occupancy factors in the average unit cell, as in the melilite phases.^8^ In fact, the presence of partial together with mixed site occupancies in the average unit cell of the melilite phases necessitates computations on a configurational ensemble, thereby going beyond the use of a single configuration. Hence, the NMR parameters were computed by considering a broad range of local structures to model the site disorder in the average unit cell. In particular, calculations were performed on the complete set of symmetrically inequivalent configurations determined with the SOD program, thereby enabling an exhaustive modeling of both mixed La^3+^/Sr^2+^ occupancies in the cationic layer and partial O4 site occupancies of the interstitial oxygen, the latter associated with a change in coordination number of the nearest Ga sites, as discussed below.

**Figure 3:**
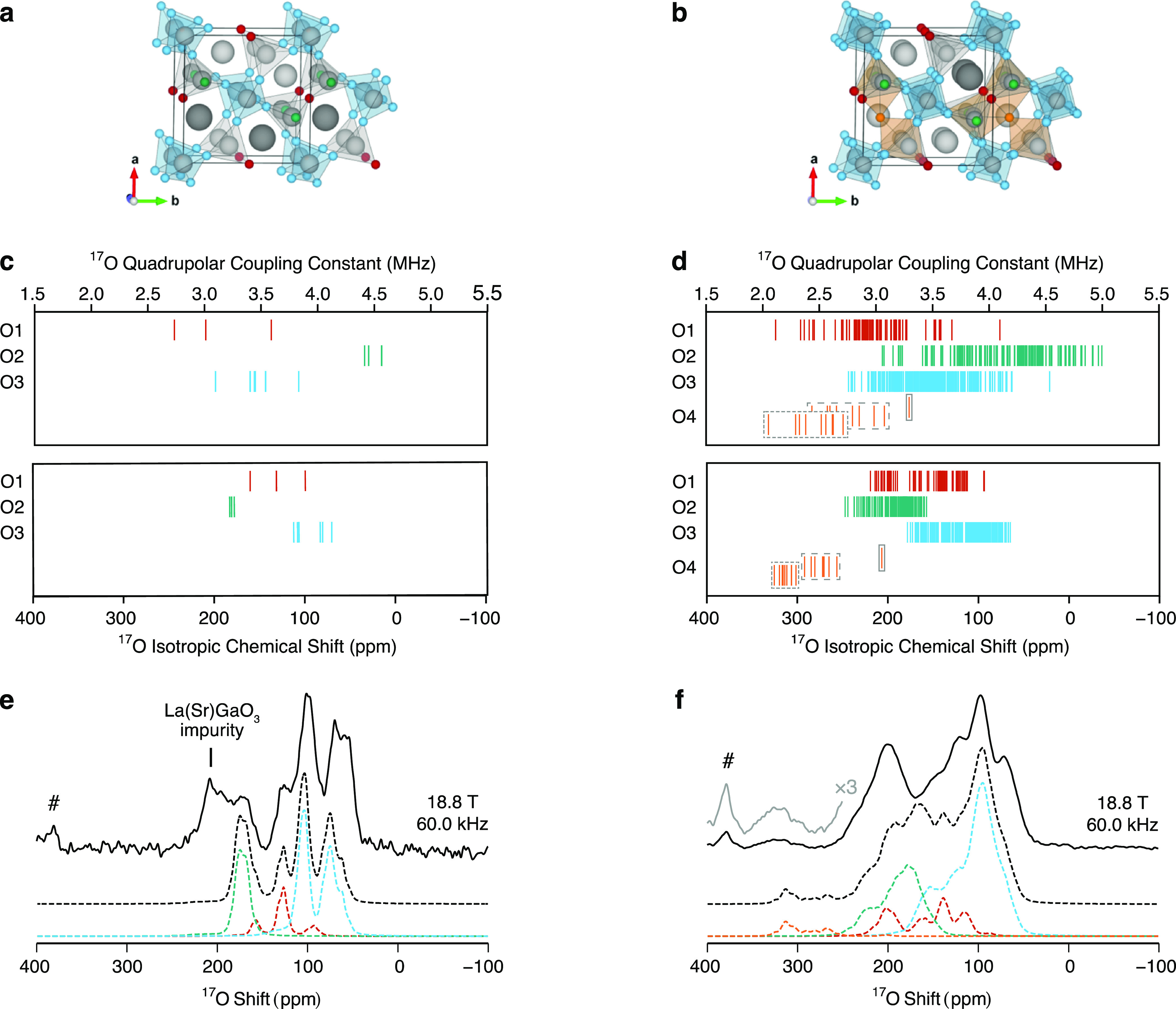
Examples of (a) LaSrGa_3_O_7_ and (b) La_1.5_Sr_0.5_Ga_3_O_7.25_ configurations determined with the SOD approach^34^ and optimized with DFT highlighting the O1 (red), O2 (green), O3 (blue), and O4 (orange) crystallographically distinct oxide ions as well as the Ga1O_4_ (blue tetrahedra), Ga2O_4_ (gray tetrahedra), and Ga2′O_5_ (orange polyhedra) units. The cells are expanded to show the pentagonal rings, and the Sr atoms obscuring the O4 sites along the *c*-axis are removed for visual purposes. ^17^O isotropic chemical shifts and quadrupolar coupling constants of (c) LaSrGa_3_O_7_ and (d) La_1.5_Sr_0.5_Ga_3_O_7.25_ computed with the GIPAW approach^33^ on a set of symmetrically inequivalent configurations and grouped according to the crystallographically distinct site. The values corresponding to O4 in the proximity of a pair of Sr^2+^/Sr^2+^, La^3+^/Sr^2+^, or La^3+^/La^3+^ cations are surrounded by solid, dashed, or dotted boxes, respectively. ^17^O MAS NMR spectra of (e) LaSrGa_3_^17^O_7_ and (f) La_1.54_Sr_0.46_Ga_3_^17^O_7.27_ at 18.8 T with a MAS rate of ν_r_ = 60.0 kHz. The total simulated spectra (dashed black lines) below the experimental data (solid black lines) are obtained from the computed NMR parameters. The contribution to the total simulated spectrum of each crystallographically distinct site (colored dashed lines) is color-coded in line with the oxide ions shown in the structure. The hash symbol (#) indicates the signal of the ZrO_2_ rotor. A magnified view (with ×3 intensity) of the O4 signal is shown in gray.

Figure 3e,f shows ^17^O MAS NMR spectra of LaSrGa_3_O_7_ and La_1.54_Sr_0.46_Ga_3_O_7.27_ simulated at 18.8 T with a MAS rate ν_r_ = 60.0 kHz from the ^17^O NMR parameters (Figure 3c,d) computed using the GIPAW method^33,52^ on a set of 2 and 18 symmetrically inequivalent configurations which were generated with SOD, respectively, starting from a LaSrGa_3_O_7_ unit cell and a La_1.5_Sr_0.5_Ga_3_O_7.25_ 1 × 1 × 2 super cell and subsequently optimized using DFT,^47^ as described in the Experimental Section. Illustrative examples of symmetrically inequivalent configurations obtained for LaSrGa_3_O_7_ and La_1.5_Sr_0.5_Ga_3_O_7.25_ which present explicit distributions of the atoms among the sites characterized by mixed and partial occupancies are shown in Figure 3a,b, while the complete ensemble of structures used in this analysis is presented in Figure S6. The interstitial defects were incorporated into the framework in the O4 position close to the center of the pentagonal ring formed by the five nearest Ga sites, in accordance with the structural model proposed based on neutron diffraction experiments^8,16^ and confirmed by DFT calculations.^17^

The simulated ^17^O MAS NMR spectra shown in Figure 3e,f (LaSrGa_3_O_7_ and La_1.5_Sr_0.5_Ga_3_O_7.25_, respectively) are the sum of the ^17^O MAS NMR spectra of each symmetrically inequivalent configuration weighted by the configurational degeneracy only (i.e., in the high-temperature limit *e*^–Δ*E*/*k*_B_*T*^ → 1 also referred to as the limit of full disorder). In fact, close agreement between the experimental and computational results is obtained when considering the configurations as equally accessible, suggesting a random La^3+^/Sr^2+^ ordering likely caused by the elevated temperatures used in the synthesis procedure and indicating that a configurational equilibrium is not reached when the samples are cooled to ambient conditions (Figure S7). A clear distinction between the *C*_Q_ and δ_iso,cs_ values calculated for the bridging O1 and O3, and nonbridging O2 oxide ions in LaSrGa_3_O_7_ is revealed, with larger values observed for the latter. Overall, the excellent agreement between the simulated and experimental ^17^O MAS NMR spectra of LaSrGa_3_O_7_ enabled the computationally guided NMR spectral assignment as highlighted in Table 1 and Figure 3. The comparison between the simulated and experimental ^17^O MAS NMR spectra of LaSrGa_3_O_7_ also reveals an inhomogeneous ^17^O enrichment which favors the enrichment of the bridging oxide ions O1 and O3 and reflects the poor ionic transport properties of the LaSrGa_3_O_7_ phase. A similar trend in the distribution of the NMR parameters computed for the bridging O1 and O3 and nonbridging O2 oxide ions in La_1.5_Sr_0.5_Ga_3_O_7.25_ is observed, even though the values are scattered over a larger range, in accordance with the experimental results. While the spectra obtained for the La^3+^-doped phase match to a lower extent due to the enhanced disorder which results in significantly overlapping signals (Figure 3f), it can be concluded that (i) the strongly deshielded resonance observed in the experimental data corresponds to the interstitial oxide ions located in the O4 position, (ii) the nonbridging oxide ions O2 extensively contribute to the resonance at δ of ∼203 ppm and (iii) the signals in the 30–170 ppm region can be mainly assigned to the bridging oxide ions O1 and O3. This spectral assignment is also supported by the relative area of the deconvoluted experimental signals assigned to the bridging O1/O3, nonbridging O2, and interstitial O4 ions (approximately 5:2.3:0.25) which is in excellent agreement with the expected values (5:2:0.27). These results are also indicative of quantitative ^17^O enrichment of the La_1.54_Sr_0.46_Ga_3_^17^O_7.27_ sample and ^17^O scrambling across all sites, as discussed below in the oxygen dynamics section. In the configurational ensemble, the interstitial oxide ions can be surrounded by either Sr^2+^/ Sr^2+^, Sr^2+^/La^3+^, or La^3+^/La^3+^ pairs in the cationic layer (Figure S6) and the computed ^17^O NMR parameters for O4 indicate that the predicted isotropic chemical shift increases while the quadrupolar coupling constant decreases with increasing La^3+^ content in its proximity.

**Figure 4:**
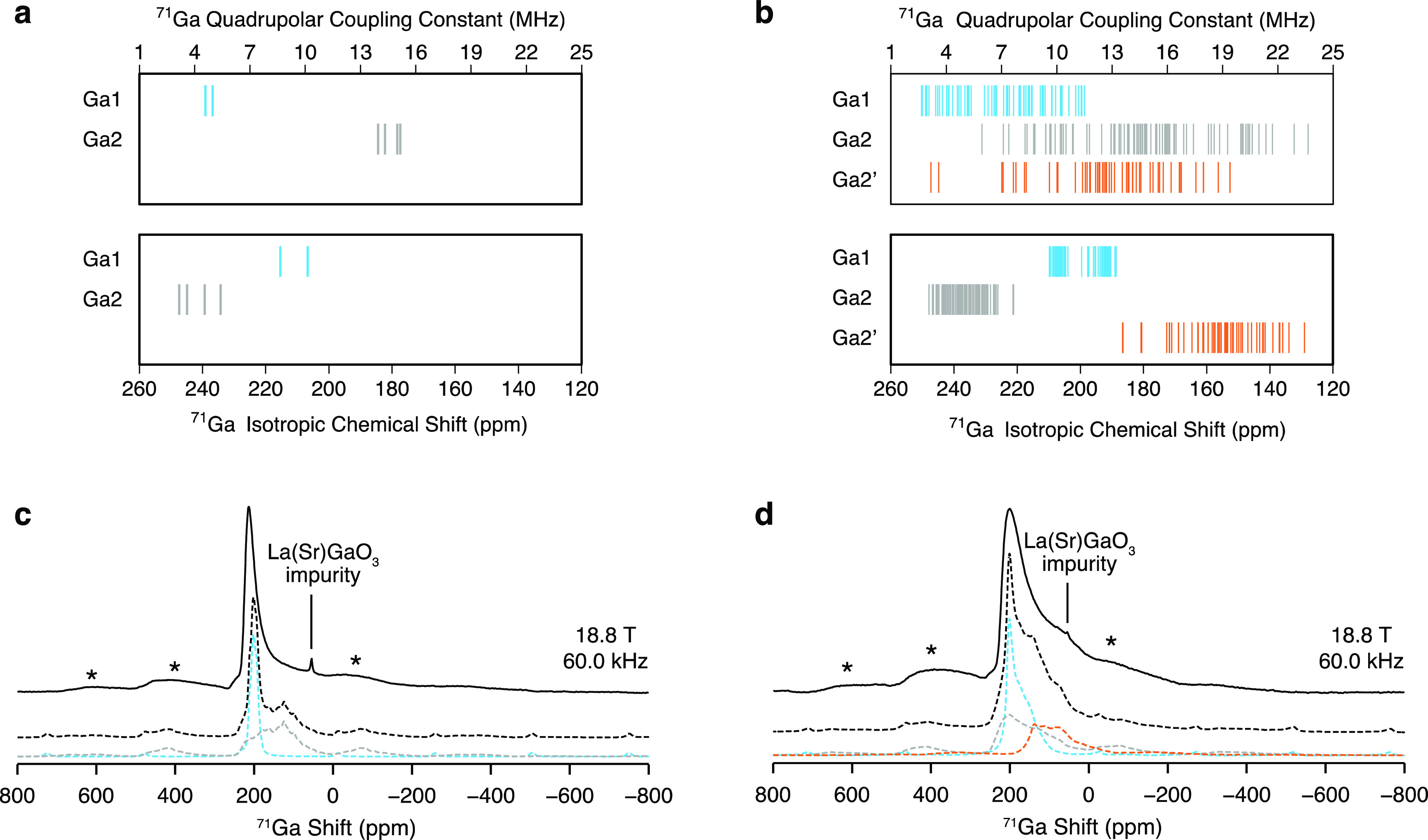
^71^Ga isotropic chemical shifts and quadrupolar coupling constants of (a) LaSrGa_3_O_7_ and (b) La_1.5_Sr_0.5_Ga_3_O_7.25_ computed with the GIPAW approach^33^ on a set of symmetrically inequivalent configurations determined with the SOD approach^34^ for LaSrGa_3_O_7_ and La_1.5_Sr_0.5_Ga_3_O_7.25_ and grouped according to the crystallographic distinct sites Ga1 (blue), Ga2 (gray), and Ga2′ (orange). ^71^Ga Hahn echo MAS NMR spectra of (c) LaSrGa_3_O_7_ and (d) La_1.54_Sr_0.46_Ga_3_O_7.27_ at 18.8 T with a MAS rate ν_r_ = 60.0 kHz, also presenting a small signal corresponding to a La(Sr)GaO_3_ impurity. The total simulated spectra (dashed black lines) below the experimental data (solid black lines) are obtained from the computed NMR parameters. The colorful dashed lines indicate the contribution of the crystallographically distinct Ga sites to the total simulated spectrum. The asterisks (*) denote the spinning sidebands.

The presence of excess oxide ions is stabilized by the ability of the Ga sites to increase their coordination number, and ^71^Ga (and ^69^Ga) MAS NMR represents a possible approach to detect these changes owing to the well-known relationship, valid for a wide range of cations, associating the lowering of the isotropic chemical shift to an increase in the coordination number.^31,54,64^^139^La and ^87^Sr NMR experiments are in principle suitable to gain insight into the La^3+^/Sr^2+^ site disorder but were not performed in this work as discussed in the Supporting Information. Although Ga has two NMR-active nuclei, ^71^Ga (*I* = 3/2) is regarded to be the nucleus of choice because it is more sensitive and gives rise to signals narrower than those of ^69^Ga (also *I* = 3/2). While ^71^Ga MAS NMR spectra of LaSrGa_3_O_7_ have been previously documented,^65^ high-field ^71^Ga data were yet to be acquired for the La^3+^-doped melilite. The ^71^Ga Hahn echo MAS NMR experiments of the LaSrGa_3_O_7_ and La_1.54_Sr_0.46_Ga_3_O_7.27_ melilite samples recorded with a MAS rate of ν_r_ = 60.0 kHz are shown in Figure 4c,d and present signals severely affected by second-order quadrupolar effects. One intense signal at δ ∼ 214 ppm which exhibits a tail at lower frequencies typical of line shapes originating from Czjzek-distributed quadrupolar parameters^66^ is observed for LaSrGa_3_O_7_ and assigned to a 4-coordinate Ga site. The spectrum of La_1.54_Sr_0.46_Ga_3_O_7.27_ displays a similar spectral line shape which, however, is characterized by a more pronounced shoulder at δ of ∼90 ppm (i.e., δ_iso,cs_ of ∼150 ppm assuming *C*_Q_ = 12 MHz and η_Q_ = 0), suggesting the presence of a 5-coordinate site on the basis of NMR parameters computed for other 5-coordinate Ga sites, such as LaGaGe_2_O_7_ (δ_iso,cs_ = 90 ppm and *C*_Q_ = 12 MHz),^54^ Sr- and Mg-doped LaGaO_3_ (δ_iso,cs_ = 150 ppm and *C*_Q_ = 10 MHz),^31^ La_3_Ga_4_Ge_2_O_14.5_ (δ_iso,cs_ = 177 ppm and *C*_Q_ = 10 MHz as well as δ_iso,cs_ = 109 ppm and *C*_Q_ = 23 MHz),^10^ and La_2_Ga_3_O_7.5_ (δ_iso,cs_ = 150 ppm and *C*_Q_ = 13 MHz).^67^

The nontrivial spectral line shapes observed in the ^71^Ga MAS NMR spectra limit the amount of information that can be unambiguously deduced, and the computed ^71^Ga NMR parameters (Figure 4a,b) are pivotal to decode the structural details entailed in the experimental spectra. As mentioned above, the interstitial defects O4 are located close to the center of the pentagonal ring formed by the five nearest Ga sites, two Ga1 and three Ga2 sites. Based on the optimized coordinates of all symmetrically inequivalent configurations, the two Ga1 sites are located at a further distance (>3 Å) from O4 than the remaining three Ga2 sites (<2.5 Å) which are therefore believed to change coordination number and become 5-coordinate Ga2′ sites (Figure S8). Furthermore, it is observed that in all symmetrically inequivalent configurations, the O4 interstitial ion is closer to the Ga2′ site located between the two Ga1 sites than the remaining two Ga2′ sites adjacent to each other in the pentagonal ring. A clear distinction between the δ_iso,cs_ values predicted for the different Ga sites in both LaSrGa_3_O_7_ and La_1.5_Sr_0.5_Ga_3_O_7.25_ is observed, with δ_iso,cs_ of Ga1, Ga2, and Ga2′, respectively, within the 185–215, 220–250, and 126–185 ppm range. The substantial difference in δ_iso,cs_ predicted for the Ga2 and Ga2′ sites supports that the three nearest Ga sites surrounding the oxide ions have 5-coordinate character, as suggested above, and the computational modeling is consistent with the intensity of the ^71^Ga resonances observed in the experimental spectra. For LaSrGa_3_O_7_, *C*_Q_ values corresponding to Ga1 ranging from 4.5 to 5 MHz are determined, while significantly larger quadrupolar coupling constants between 14 and 15.5 MHz are computed for Ga2, in alignment with the pronounced distortion of the Ga2 tetrahedral site. The *C*_Q_ values predicted for La_1.5_Sr_0.5_Ga_3_O_7.25_ follow a similar trend, yet they are distributed over a significantly larger interval, reflecting enhanced structural disorder in the material. For the Ga2′ site, *C*_Q_ values in the 3–20 MHz range are computed. Figure S9a,b reports a comparison of the δ_iso,cs_, δ_QIS_ (deduced from the *C*_Q_ and η_Q_ as in eqs 7 and 8) and δ computationally predicted for LaSrGa_3_O_7_ and La_1.5_Sr_0.5_Ga_3_O_7.25_. From the comparison between the experimental and predicted ^71^Ga MAS NMR spectra (Figure 4c,d), we conclude that (i) the relatively sharp signal at a shift δ ∼ 214 ppm corresponds to the Ga1 tetrahedral site, (ii) the tail observed at higher frequencies originates from the presence of a distorted Ga2 tetrahedral site associated with a significantly larger quadrupolar coupling constant, and (iii) the shoulder observed in the spectrum of the La-doped phase corresponds to a 5-coordinate Ga2′ site which forms to accommodate the interstitial oxide ions. While the chemical shift anisotropy tensor has been included in the simulation of the ^71^Ga MAS NMR spectra, this contribution is found to be negligible compared to the significantly stronger quadrupolar interactions, as shown in Figure S10a–f. The large *C*_Q_ values predicted for Ga2 and Ga2′ challenge the detection of these broad signals in the ^71^Ga 3QMAS experiments (Figures S11a,b and S12a,b), as discussed in the Supporting Information. The ^71^Ga quadrupolar NMR parameters obtained for Ga1 from the 3QMAS spectra as detailed in the Experimental Section are listed in Table 1.

### Oxygen Dynamics

**Figure 5:**
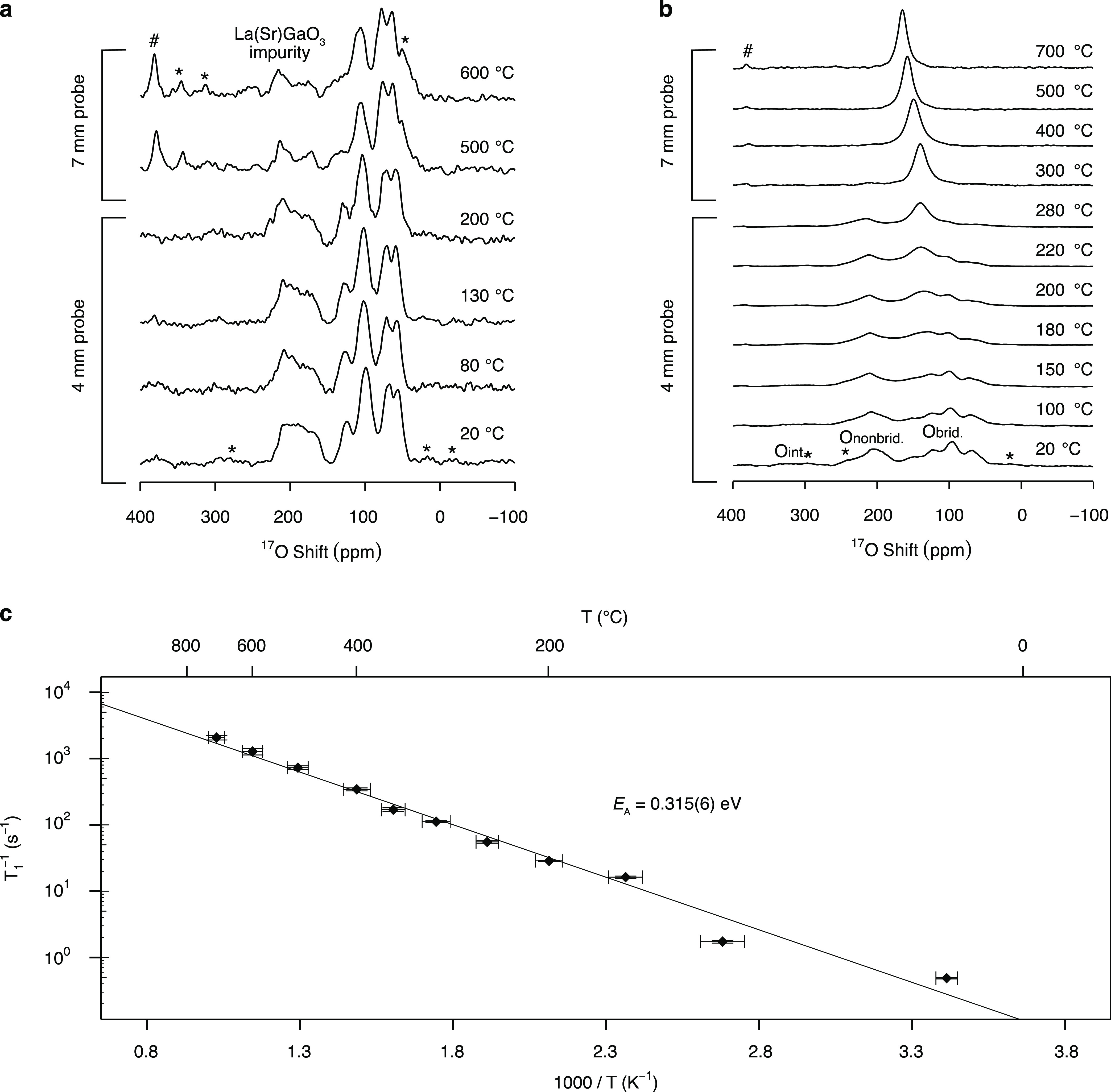
High-temperature ^17^O MAS NMR spectra of (a) LaSrGa_3_^17^O_7_ and (b) La_1.54_Sr_0.46_Ga_3_^17^O_7.27_ recorded at 20 T with either a 4 mm MAS probe with a MAS rate of ν_r_ = 10.0 kHz or a 7 mm laser-heated MAS probe with a MAS rate of ν_r_ = 4.0 kHz. The hash symbol (#) and asterisks (*) denote the signal of the ZrO_2_ rotor and the spinning sidebands, respectively. Signals assigned to the interstitial (O_int._), nonbridging (O_nonbrid._), and bridging (O_brid._) oxide ions in La_1.54_Sr_0.46_Ga_3_O_7.27_ are highlighted. (c) ^17^O spin–lattice relaxation rates as a function of reciprocal temperature of La_1.54_Sr_0.46_Ga_3_^17^O_7.27_ recorded at 20 T with the 7 mm laser-heated MAS probe, highlighting the activation energy *E*_A_ of the short-range motion.

Selected ^17^O high-temperature MAS NMR spectra of LaSrGa_3_^17^O_7_ and La_1.54_Sr_0.46_Ga_3_^17^O_7.27_ recorded up to 700 °C at 20 T are presented in Figure 5a,b with the complete data set shown in Figure S13a,b. ^17^O MAS NMR experiments in the 20–300 °C range were performed with a 4 mm high-temperature probe, while data from 300 to 700 °C were recorded using a 7 mm laser-heated MAS probe. Notably, the ^17^O NMR spectra of LaSrGa_3_^17^O_7_ are largely not affected by the increase in temperature up to 600 °C, indicating that the oxide ions are very likely not involved in dynamic processes occurring at a rate  in the order of the frequency separation Δν between the ^17^O resonances (i.e., ∼3 kHz < τ^*–*1^ < ∼31 kHz), in accordance with the poor oxide ion conductivity measured for the undoped phase.^8^ This is in very sharp contrast with the significant changes in the position and line shape of the ^17^O signals observed in the ^17^O NMR spectra of La_1.54_Sr_0.46_Ga_3_^17^O_7.27_ collected up to 700 °C, reflecting the remarkably high oxide ion conductivity of this phase.^8^ The room temperature ^17^O MAS NMR spectra of La_1.54_Sr_0.46_Ga_3_^17^O_7.27_ recorded before and after heating to 700 °C are largely unchanged (Figure S14) and suggest that reversible changes are detected upon heating. Very importantly, all of the ^17^O resonances in the spectra coalesce into a single peak above 300 °C, implying that the incorporation of interstitial defects into the framework leads to enhanced ionic motion and strongly indicating the occurrence of chemical exchange between all of the oxide ions at a rate in the order of the frequency separation between the spectral features in the absence of chemical exchange, i.e., up to τ^–1^ ∼ 50 kHz, at the coalescence temperature of ∼300 °C. These data are in strong agreement with the scrambling of ^17^O across all oxygen environments at the isotopic enrichment temperature (i.e., 750 °C) that gives rise to an experimental ^17^O MAS NMR spectrum at room temperature capturing the relative concentration of all oxygen environments, as discussed above, and support an indirect interstitial mechanism for the oxygen ion conduction involving all oxide ions.

The spectral centroid of the 0–350 ppm region of the ^17^O high-temperature MAS NMR spectra of La_1.54_Sr_0.46_Ga_3_^17^O_7.27_ is observed to linearly increase with temperature at a rate of 0.029 ppm/°C (i.e., 20 ppm difference between 20 and 700 °C), as highlighted in Figure S13a,b, likely reflecting the measured thermal expansion of the cell parameters^8^ and the lowering of the quadrupolar coupling constant with increasing temperature. Closer inspection of Figures 5b and S13a,b reveals that the signal(s) between 170 and 260 ppm predominantly corresponding to the nonbridging oxide ions O2 coalesce into a single symmetric peak at 180 °C and the position of the maximum intensity of these resonances shifts from 200 ppm at 20 °C to 220 ppm at 280 °C, although their weighted average remains constant up to 280 °C. The signals in the 110–170 ppm region begin to broaden at 80 °C to then coalesce at 180–200 °C into a single peak at a shift δ of ∼135 ppm. Additionally, the line width of the resonances between 30 and 110 ppm broadens above 180 °C until all signals at chemical shifts δ < 170 ppm merge into a single broad signal at a shift δ of ∼140 ppm above 280 °C. These results indicate that the bridging oxide ions O1 and O3 exchange at a rate up to τ^–1^ of ∼22 kHz at the coalescence temperature of 180–220 °C.

The interstitial oxide ions O4 are most likely involved in this motional process based on (i) the critical role they play in increasing the ionic conductivity in melilite,^8^ (ii) the NMR observation of local oxygen dynamics in La_1.54_Sr_0.46_Ga_3_O_7.27_ which is not detected for LaSrGa_3_O_7_ (Figure 5a and b) and (iii) the proximity of the O4 ions to the O1 and O3 sites in the unit cell. However, the detection of the O4 signal is challenged by the small percentage of interstitial defects in La_1.54_Sr_0.46_Ga_3_^17^O_7.27_ resulting in a weaker signal at δ_iso,cs_ of ∼325 ppm combined with the unfavorable Boltzmann population difference at high temperature and the partial overlap of this resonance with spinning sidebands. Although the considerable structural disorder in La_1.54_Sr_0.46_Ga_3_O_7.27_ hinders the extraction of more detailed quantitative information from the high-temperature ^17^O MAS NMR spectra, the data supports onset of chemical exchange (τ^–1^ up to ∼22 kHz) among bridging oxide ions at 180–220 °C and subsequent chemical exchange (τ^–1^ up to ∼50 kHz) above the coalescence temperature (∼300 °C) involving all oxygen sites.

**Figure 6:**
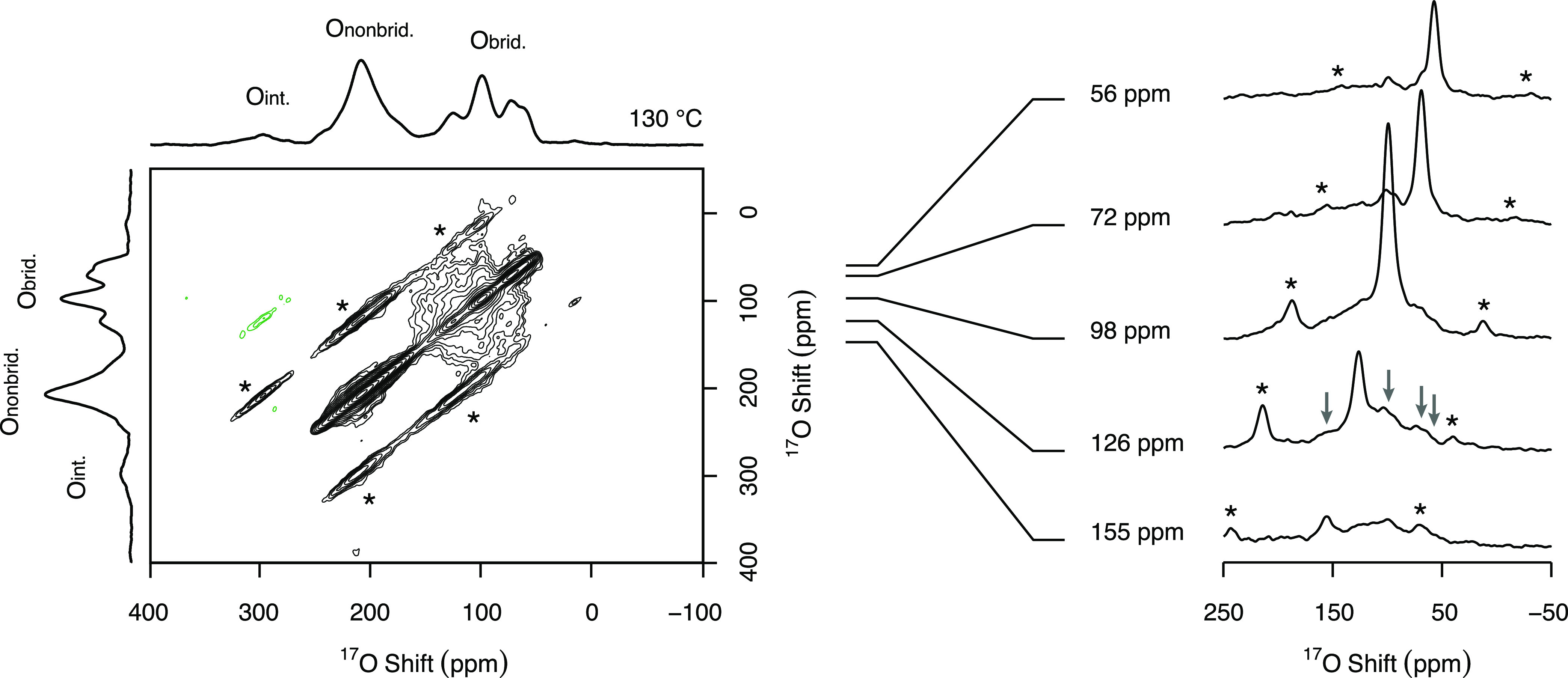
Two-dimensional ^17^O–^17^O EXSY NMR spectrum of La_1.54_Sr_0.46_Ga_3_^17^O_7.27_ recorded at 20 T at a temperature of 130 °C under a MAS rate of ν_r_ = 10.0 kHz and with a mixing time of 15 ms, highlighting signals assigned to the interstitial (O_int._), nonbridging (O_nonbrid._), and bridging (O_brid._) oxide ions. Negative peaks are shown in green, and spinning sidebands are marked with asterisks (*). Cross sections of the O_brid._ signals parallel to the *f*_2_ dimension are presented on the right. The arrows indicate the off-diagonal peaks observed in one of these cross sections.

Two-dimensional ^17^O ^17^O EXSY experiments were performed at variable temperatures and various mixing times to detect chemical exchange between the oxide ions in La_1.54_Sr_0.46_Ga_3_^17^O_7.27_. A representative spectrum recorded at 130 °C with a mixing time of 15 ms is shown in Figure 6, where the cross-peaks between the signals in the 50–170 ppm region (which do not obviously appear at a mixing time of 0 ms, as shown in Figure S15) unambiguously confirm that chemical exchange between the O1 and O3 sites occurs at this temperature. Owing to the reduced intensity of the cross-peaks combined with the aforementioned absence of clearly distinct signals, mainly qualitative data can be extracted from the ^17^O–^17^O EXSY experiments. Nevertheless, these results are in excellent agreement with the coalescence of the ^17^O signals assigned to the bridging oxide ions at 180–220 °C observed in the high-temperature ^17^O MAS NMR spectra.

^17^O spin–lattice relaxation rates in the laboratory frame of motion *T*_1_^–1^ of LaSrGa_3_^17^O_7_ (fits shown in Figure S16) and La_1.54_Sr_0.46_Ga_3_^17^O_7.27_ (fits shown in Figure S17) recorded at variable temperatures up to 700 °C provide insight into the dynamics in fast oxide ion conductors on the MHz time scale. While the *T*_1_^–1^ rates extracted for LaSrGa_3_^17^O_7_ are only lightly dependent on the reciprocal temperature (Figure S18), the *T*_1_^–1^ rates determined for La_1.54_Sr_0.46_Ga_3_^17^O_7.27_ increase with increasing temperature (e.g., from ∼0.5 s^–1^ at room temperature to ∼55 s^–1^ at 250 °C to ∼700 s^–1^ at 500 °C, as shown in Figure S17), suggestive of short-range ionic motion on the MHz time scale (Figure 5c). The linear dependence of ln (*T*_1_^–1^) vs *T*^–1^ is indicative of the detection of thermally activated short-range ionic motion which can be fitted to Arrhenius behavior (eq 9) to extract the activation energy of the short-range motion, yielding *E*_A_ = 0.315(6) eV
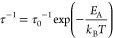
9where τ_0_^–1^ is the pre-exponential factor, *E*_A_ is the activation energy, and *k*_B_ is the Boltzmann constant. This *E*_A_ value is smaller than the long-range activation energy determined from impedance measurements^8^ (*E*_A_ = 0.42(1) and 0.85(1) eV) as NMR captures the short-range ionic motion within the temperature range detected in this work. Nevertheless, if the short-range dynamics probed with solid-state NMR leads to long-range conduction, then it can be concluded that both interstitial and framework oxide ions participate in the diffusion mechanism.

**Figure 7:**
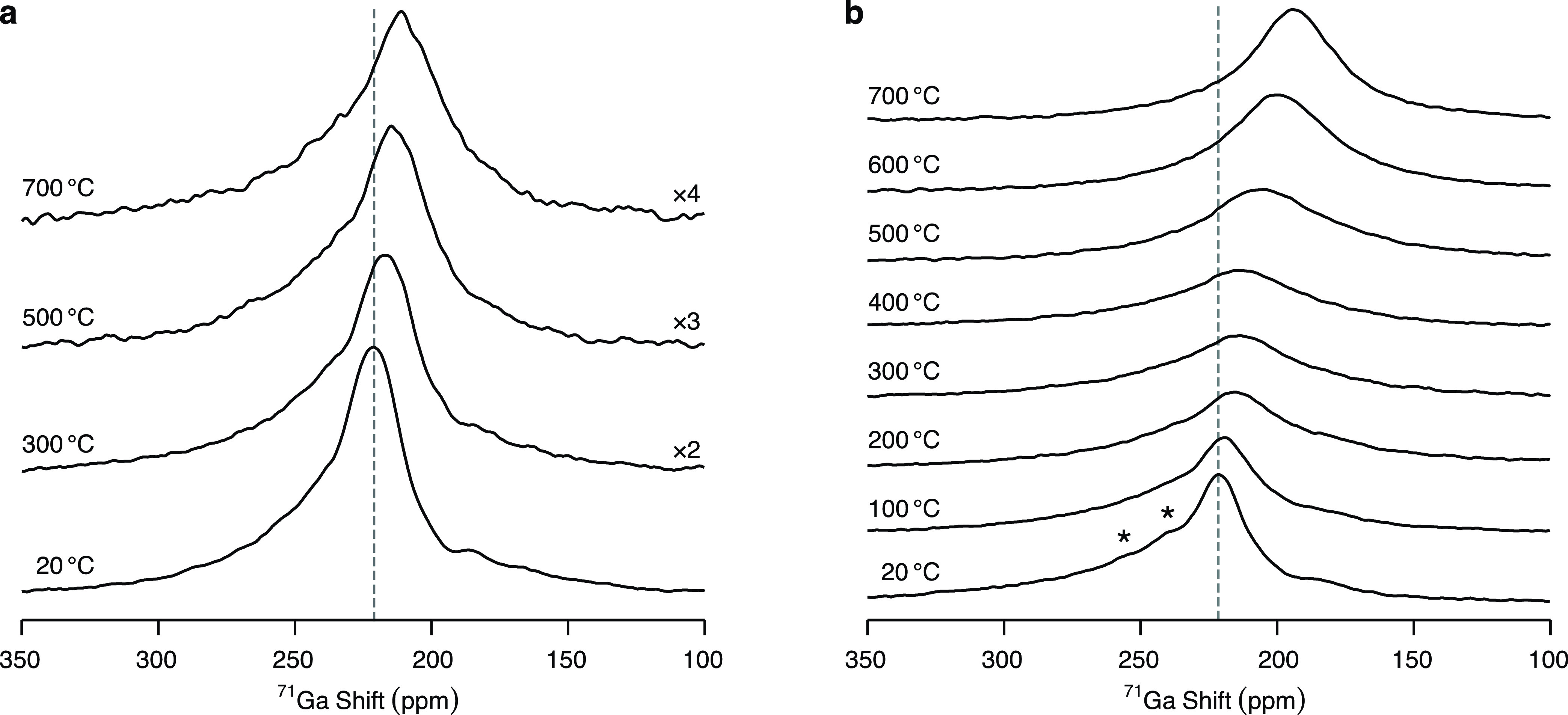
High-temperature ^71^Ga MAS NMR spectra of (a) LaSrGa_3_O_7_ and (b) La_1.54_Sr_0.46_Ga_3_O_7.27_ recorded at 20 T with a 7 mm laser-heated MAS probe under a MAS rate of ν_r_ = 4.0 kHz. The asterisks (*) denote the spinning sidebands. For visualization purposes, the spectra of LaSrGa_3_O_7_ recorded at 300, 500, and 700 °C are presented with ×2, ×3, and ×4 intensity, respectively. Visualization of changes in the line shape and position of the signals compared to the room temperature data is aided by the dashed line.

Overall, the diffusion process is likely aided by a synergic mechanism engaging all oxide ions and involving the formation and breaking of higher coordination Ga polyhedra, which rotate to facilitate the migration of O4 ions into adjacent pentagonal rings, which were originally free of interstitial. A similar diffusion pathway, although restricted to one dimension, has been observed for the fully substituted La_2_Ga_3_O_7.5_ melilite phase^67^ on the basis of MD calculations.^68^ This is further supported by the high-temperature ^71^Ga MAS NMR spectra at 20 T under an ν_r_ of 4.0 kHz presented in Figure 7a,b. The ^71^Ga resonances observed in the room temperature ^71^Ga NMR spectrum of La_1.54_Sr_0.46_Ga_3_O_7.27_ coalesce into one broad signal at approximately 400 °C which then narrows as the temperature is further increased up to 700 °C, thereby indicating the occurrence of chemical exchange. The signal assigned to the Ga1 sites shifts toward lower frequencies as the temperature increases, while only a slight shift upfield is probed in the case of LaSrGa_3_O_7_. The line broadening and narrowing of the ^71^Ga resonances observed for La_1.54_Sr_0.46_Ga_3_O_7.27_, combined with the shift of the Ga1 signal toward the 5-coordinate region of the ^71^Ga spectra, indicate that the increase in temperature leads to enhanced ionic motion around the Ga sites in La_1.54_Sr_0.46_Ga_3_O_7.27_ and reveals that the Ga sites interchange coordination environment during the ionic conduction.

## Conclusions

This work presents a combined experimental and computational NMR investigation of the configurational disorder and oxide ion diffusion mechanism in a family of melilite fast oxide ion conductors with a La_1+*x*_Sr_1–*x*_Ga_3_O_7+0.5*x*_ composition. The presence of significantly overlapping signals in the ^17^O and ^71^Ga MAS NMR spectra of the parent LaSrGa_3_O_7_ and La^3+^-doped La_1.54_Sr_0.46_Ga_3_O_7.27_ melilite phases, even under optimum experimental conditions of high field, challenges spectral assignments, which are facilitated by the computation of the NMR parameters. The NMR spectra computed considering an ensemble of symmetrically inequivalent configurations obtained with the SOD approach to model the presence of partial and mixed site occupancy of several different cationic and anionic sites in the average unit cell of the melilite phases reproduce the experimental data remarkably well, thus providing a detailed understanding of the configurational disorder and validating the ensemble-based approach to model site disorder. Importantly, interstitial oxide ions in La_1.54_Sr_0.46_Ga_3_O_7.27_ were identified in the corresponding ^17^O MAS NMR spectrum and the presence of a 5-coordinate Ga site which originates to accommodate these interstitials was successfully observed in the ^71^Ga MAS NMR spectrum.

^17^O and ^71^Ga high-temperature MAS NMR experiments up to 700 °C were performed to gain insight into the local dynamics in La_1.54_Sr_0.46_Ga_3_O_7.27_ and to establish the oxide ion diffusion pathway. Coalescence of all of the ^17^O signals and ^71^Ga in the corresponding high-temperature MAS NMR spectra of this phase reflects the presence of extensive local motion between the oxide ions, in sharp contrast to the absence of motion observed for LaSrGa_3_O_7_. These results indicate that both interstitial and framework oxide ions are involved in the diffusion mechanism and support an indirect interstitial mechanism. This work highlights the importance of ^17^O and ^71^Ga NMR spectroscopy at high temperatures and high field to increase the understanding of oxygen dynamics in oxides and opens avenues for the design of fast ionic conductors with enhanced conduction properties.

The computation of NMR parameters with an ensemble-based approach used in conjunction with experimental NMR spectroscopy provides a powerful tool to attain detailed structural elucidation of disordered systems, especially when investigating the chemical order and disorder of elements that are not readily discernible using conventional diffraction methods. The space of symmetrically inequivalent structural models generated from ensemble-based approaches rapidly expands with the complexity of the disordered system, thereby leading to a large set of electronic structure calculations. This significantly increases the computational demand given the need for high-accuracy first-principles calculations. The current protocol is therefore expected to greatly benefit from future advances in digital chemistry that overcomes the need for such calculations, for example, by harnessing machine learning approaches to rapidly predict chemical shifts.^69,70^

## Data Availability

The research data supporting this publication are accessible from the University of Liverpool Data catalogue: https://doi.org/10.17638/datacat.liverpool.ac.uk/2229.
