# Supplementary material for: Disorder and Oxide Ion Diffusion Mechanism in La1.54Sr0.46Ga3O7.27 Melilite from Nuclear Magnetic Resonance
Source: J Am Chem Soc. 2023 Oct 2;145(40):21817–31. doi: 10.1021/jacs.3c04821 (PMC10571088; doi:10.1021/jacs.3c04821)
Supplement: Supplementary file 1 — ja3c04821_si_001.pdf [file ja3c04821_si_001.pdf]

Supplementary Materials for

Disorder and Oxide Ion Diffusion Mechanism in $La_{1.54}Sr_{0.46}Ga_3O_{7.27}$ Melilite from Nuclear Magnetic Resonance

Lucia Corti,[a,b] Dinu Iuga,[c] John B. Claridge,[a,b] Matthew J. Rosseinsky,[a,b] and Frédéric Blanc[*,a,b,d]

[a]Department of Chemistry, University of Liverpool, Liverpool L69 7ZD, United Kingdom; [b]Leverhulme Research Centre for Functional Materials Design, Materials Innovation Factory, University of Liverpool, Liverpool L69 7ZD, United Kingdom; [c]Department of Physics, University of Warwick, Coventry CV4 7AL, United Kingdom and [d]Stephenson Institute for Renewable Energy, University of Liverpool, Liverpool L69 7ZF, United Kingdom.

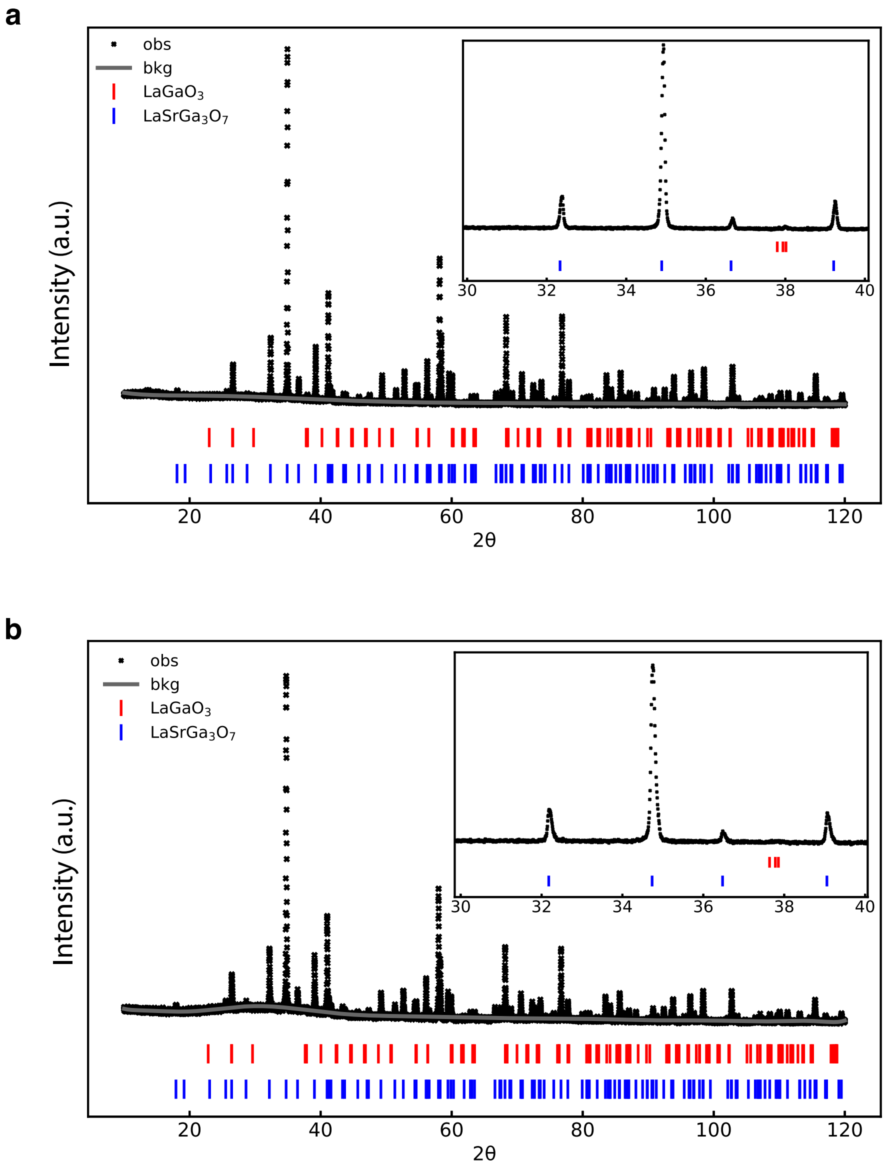

Figure S1. XRD patterns of (**a**) ^{17}O natural abundance and (**b**) ^{17}O enriched $LaSrGa_3O_7$.[1] The marks below the patterns indicate the reflections of $LaSrGa_3O_7$ (red) and $LaGaO_3$ (blue). The insets show the 30° – 40° region of the pattern.

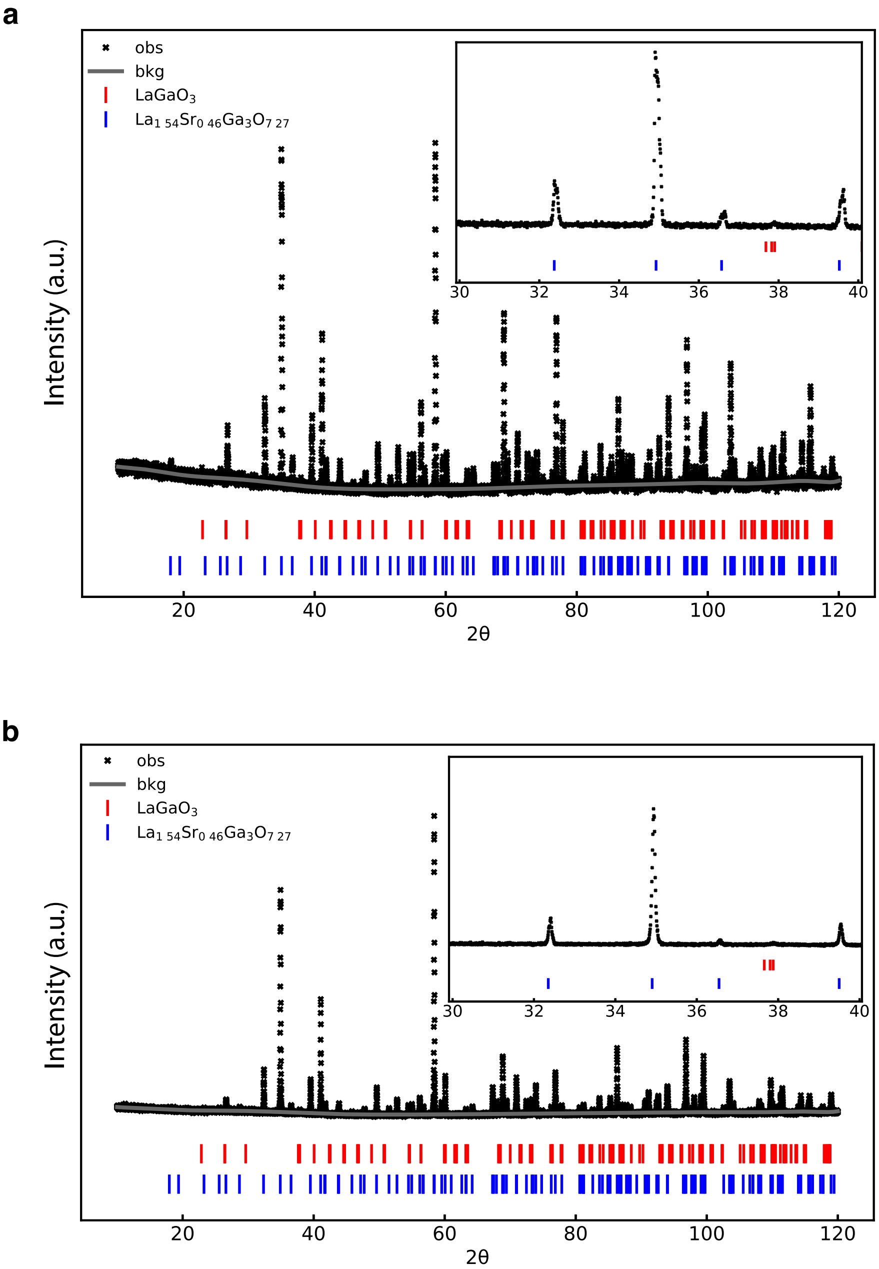

Figure S2. XRD patterns of (**a**) ^{17}O natural abundance and (**b**) ^{17}O enriched $La_{1.54}Sr_{0.46}Ga_3O_{7.27}$.[2] The marks below the patterns indicate the reflections of (red) $La_{1.54}Sr_{0.46}Ga_3O_{7.27}$ and (blue) $LaGaO_3$. The insets show the 30° – 40° region of the pattern.

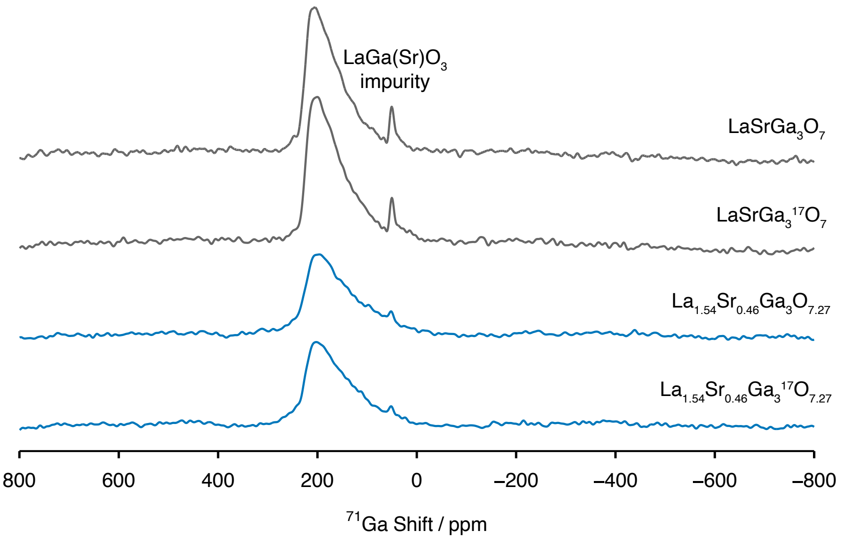

Figure S3. ^{71}Ga MAS NMR spectra of natural abundance and ^{17}O enriched $LaSrGa_3{}^{(17)}O_7$ (gray) and $La_{1.54}Sr_{0.46}Ga_3{}^{(17)}O_{7.27}$ (blue) recorded at 9.4 T with a MAS rate ν_r = 10.0 kHz, highlighting the $LaGa(Sr)O_3$ impurity.[3]

^{17}O reduced anisotropic chemical shifts of $LaSrGa_3O_7$ and $La_{1.54}Sr_{0.5}Ga_3O_{7.25}$ computed using the GIPAW approach are presented in Figure S4a-d and are comparable with the MAS rates used. The spectra shown in Figure S4e-f are predicted at the highest magnetic field strength used in this work (*i.e.*, 20 T), that is the experimental condition under which the chemical shift anisotropy interactions are the strongest and the quadrupolar interactions the weakest. A comparison between the ^{17}O spectra MAS NMR spectra with and without the chemical shift anisotropy contributions undoubtedly reveals that the latter are negligible compared to the considerably stronger quadrupolar interactions, even at the highest magnetic field of 20 T used.

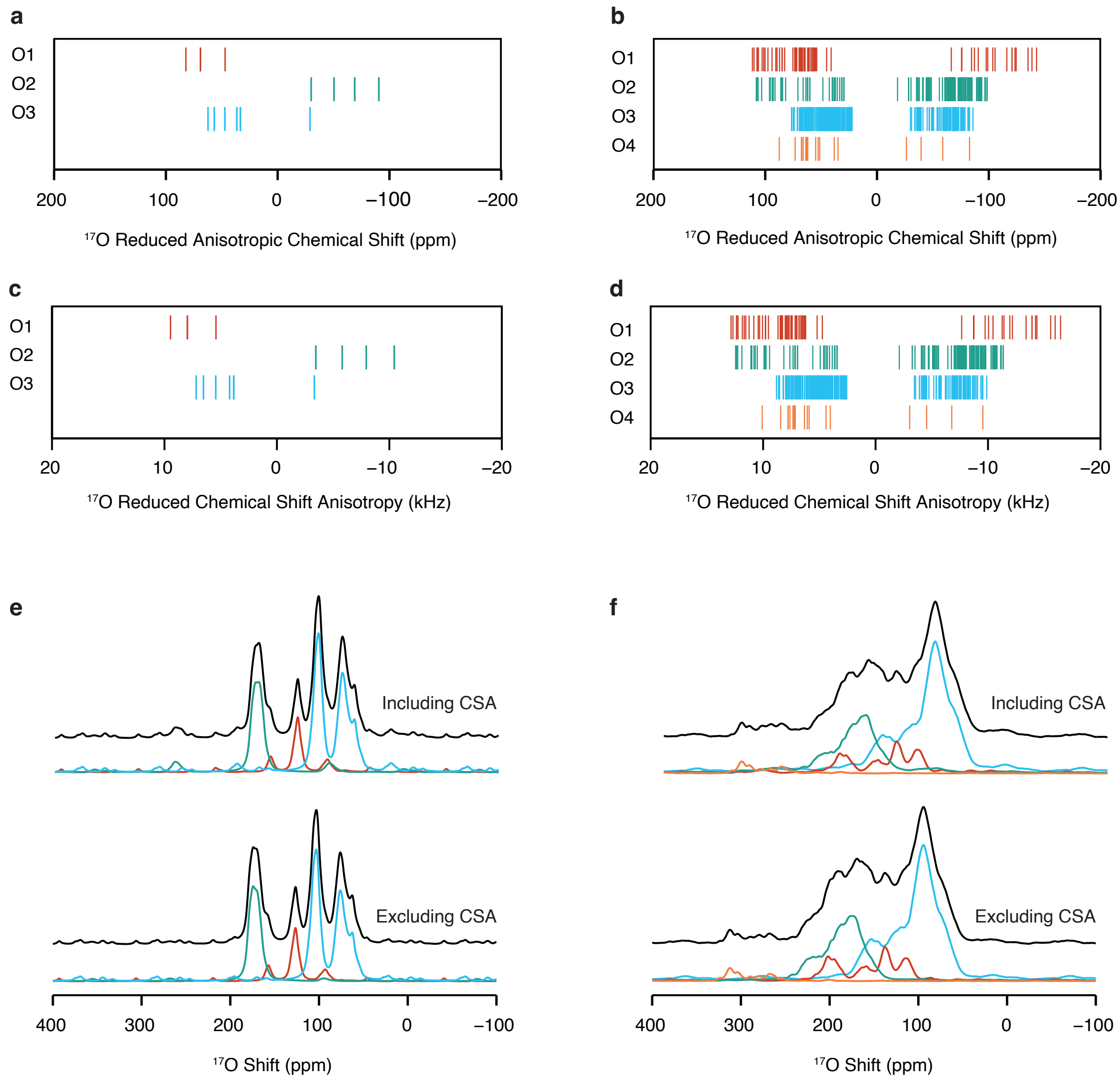

Figure S4. ^{17}O reduced chemical shift anisotropy in (**a**, **b**) ppm and in (**c**, **d**) kHz at 20 T for (**a**, **c**) $LaSrGa_3O_7$ and (**b**, **d**) $La_{1.5}Sr_{0.5}Ga_3O_{7.25}$ predicted using the GIPAW approach and grouped according to the crystallographically distinct sites O1 (red), O2 (green), O3 (blue) and O4 (orange). ^{17}O computationally predicted spectra (black lines) of (**e**) $LaSrGa_3O_7$ and (**f**) $La_{1.5}Sr_{0.5}Ga_3O_{7.25}$ simulated including (top) and excluding (bottom) the chemical shift anisotropy (CSA) contributions at 20 T and under a MAS rate $\nu_r = 10.0$ kHz. The colored lines show the contributions to the total simulated spectra from crystallographically distinct sites.

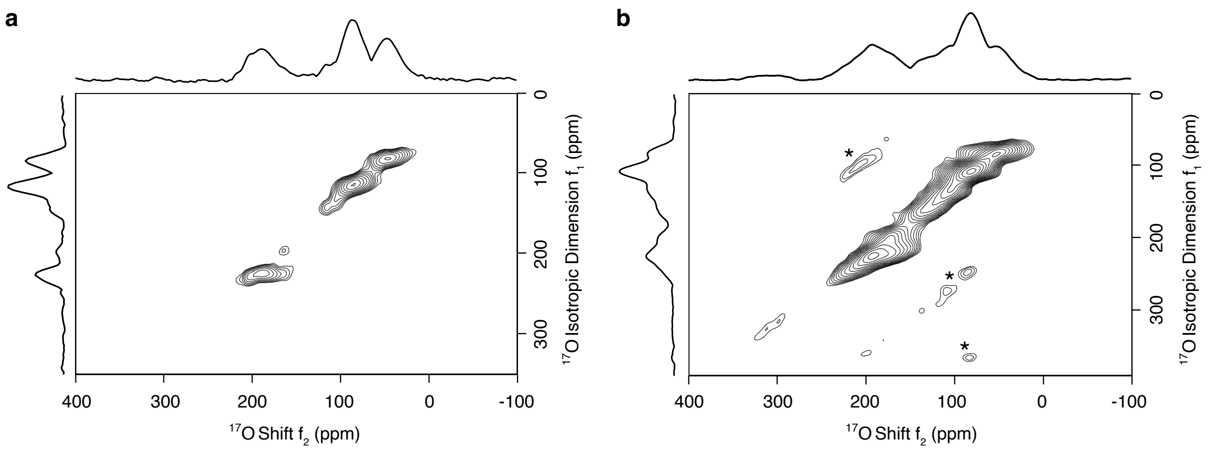

Figure S5. ^{17}O 3QMAS[4] spectra of (**a**) $LaSrGa_3{}^{17}O_7$ and (**b**) $La_{1.54}Sr_{0.46}Ga_3{}^{17}O_{7.27}$ recorded at 9.4 T with a MAS rate ν_r = 10.0 kHz.

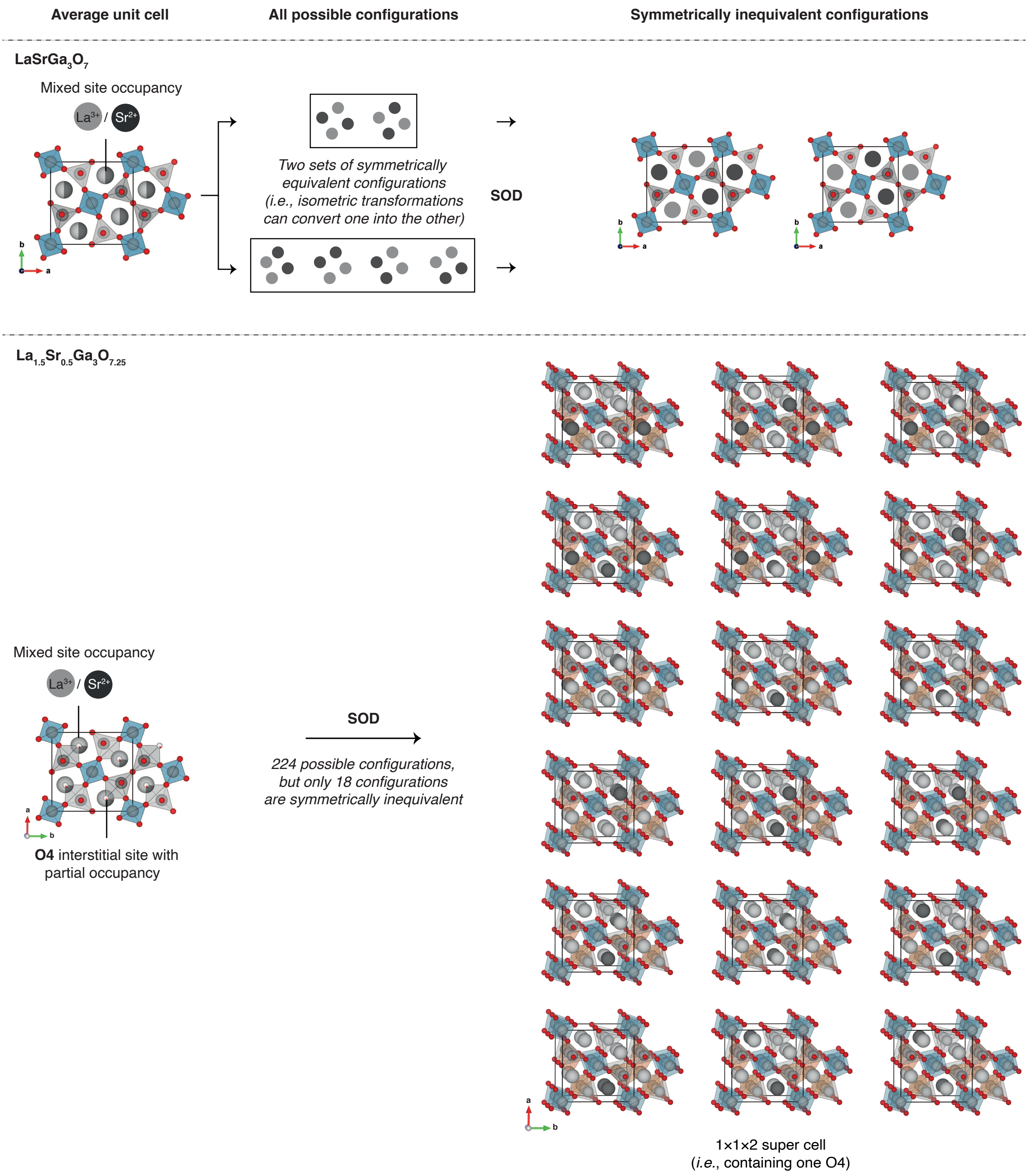

Figure S6. Schematic representation of the site occupancy disorder (SOD) approach[5] used to determine all symmetrically inequivalent configurations (right) from the site-disordered average unit cell (left). La, Sr and O atoms are shown in light gray, dark gray and red, respectively. The Ga1O4, Ga2O4 and Ga2'O5 polyhedra are presented in blue, gray and orange, respectively. The average unit cells and symmetrically inequivalent configurations are

expanded to show the pentagonal rings formed by the five Ga sites surrounding the interstitial oxide ion. The figure schematically highlights the 6 possible ways in which the La^{3+} and Sr^{2+} cations can be distributed in the $LaSrGa_3O_7$ unit cell (center) and the two symmetrically inequivalent distributions (right) which were used to calculate the $LaSrGa_3O_7$ NMR parameters. Considering the 1×1×2 super cell, the total number of configurations for $La_{1.5}Sr_{0.5}Ga_3O_{7.25}$ is 224^6 (not shown in the figure for simplicity), but only 18 configurations (right) are symmetrically inequivalent and constitute the ensemble of $La_{1.5}Sr_{0.5}Ga_3O_{7.25}$ structures used in this work.

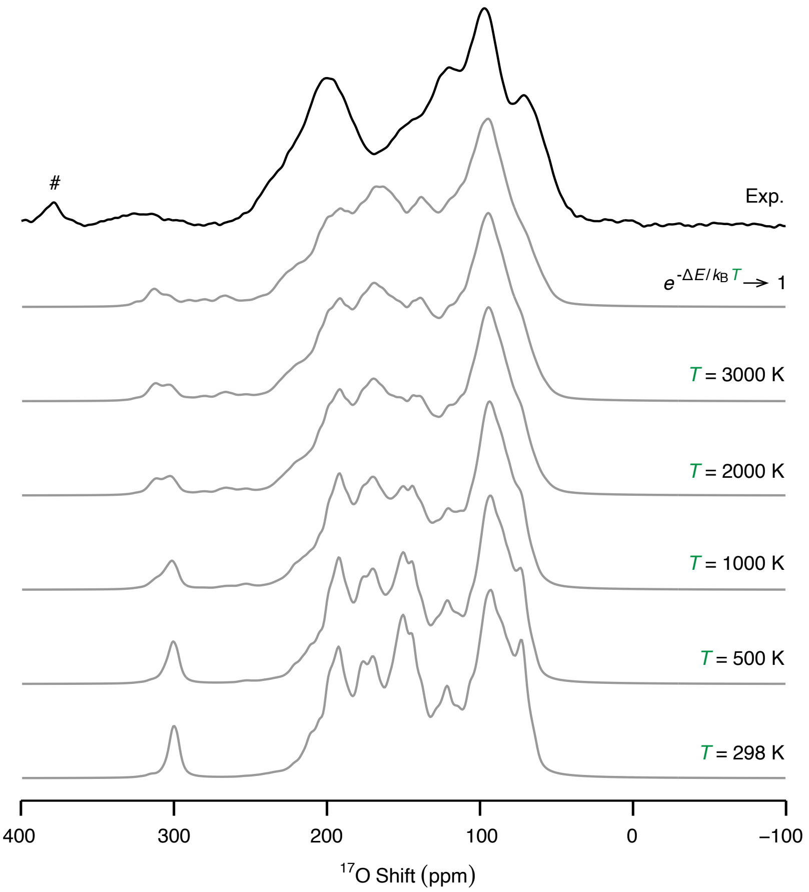

Figure S7. ^{17}O MAS NMR spectrum of $La_{1.54}Sr_{0.46}Ga_3{}^{17}O_{7.27}$ recorded at 18.8 T with a MAS rate ν_r = 60.0 kHz. The simulated spectra (gray) shown below the experimental data (black) are obtained by weighting the spectrum of each configuration by an additional Boltzmann factor at temperature T = 298 K, 500 K, 1000 K, 2000 K and 3000 K as well as in the limit of full disorder (*i.e.*, $e^{-\Delta E/k_B T} \rightarrow 1$). The hash symbol (#) indicates the ^{17}O signal of the ZrO_2 rotor.

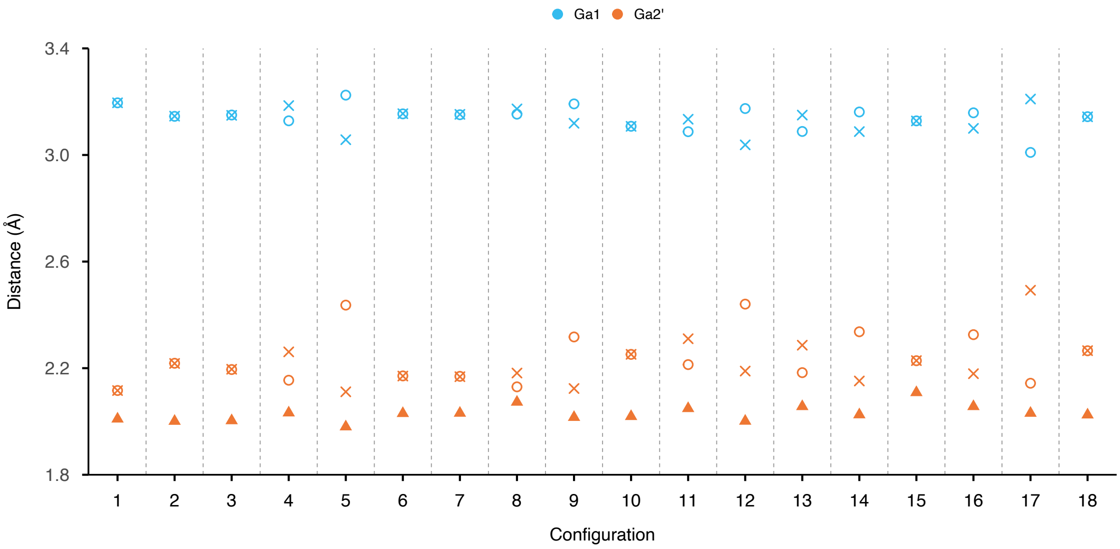

Figure S8. Distances between the interstitial oxide ion and the five Ga sites in the pentagonal ring in each symmetrically inequivalent configuration of $La_{1.5}Sr_{0.5}Ga_3O_{7.25}$. The two Ga1 sites are shown in blue and the three Ga2' sites in orange.

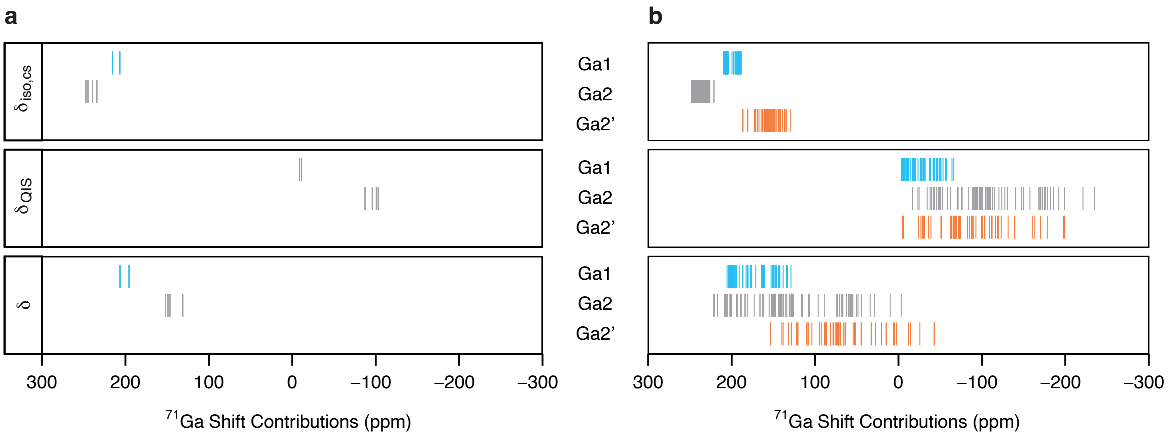

Figure S9. (**a**) $LaSrGa_3O_7$ and (**b**) $La_{1.5}Sr_{0.5}Ga_3O_{7.25}$ ^{71}Ga isotropic chemical shifts ($\delta_{iso,cs}$), quadrupolar induced shifts (δ_{QIS}) and shifts (δ) at 18.8 T predicted using the GIPAW approach and grouped according to the crystallographically distinct sites Ga1 (blue), Ga2 (gray) and Ga2' (orange).

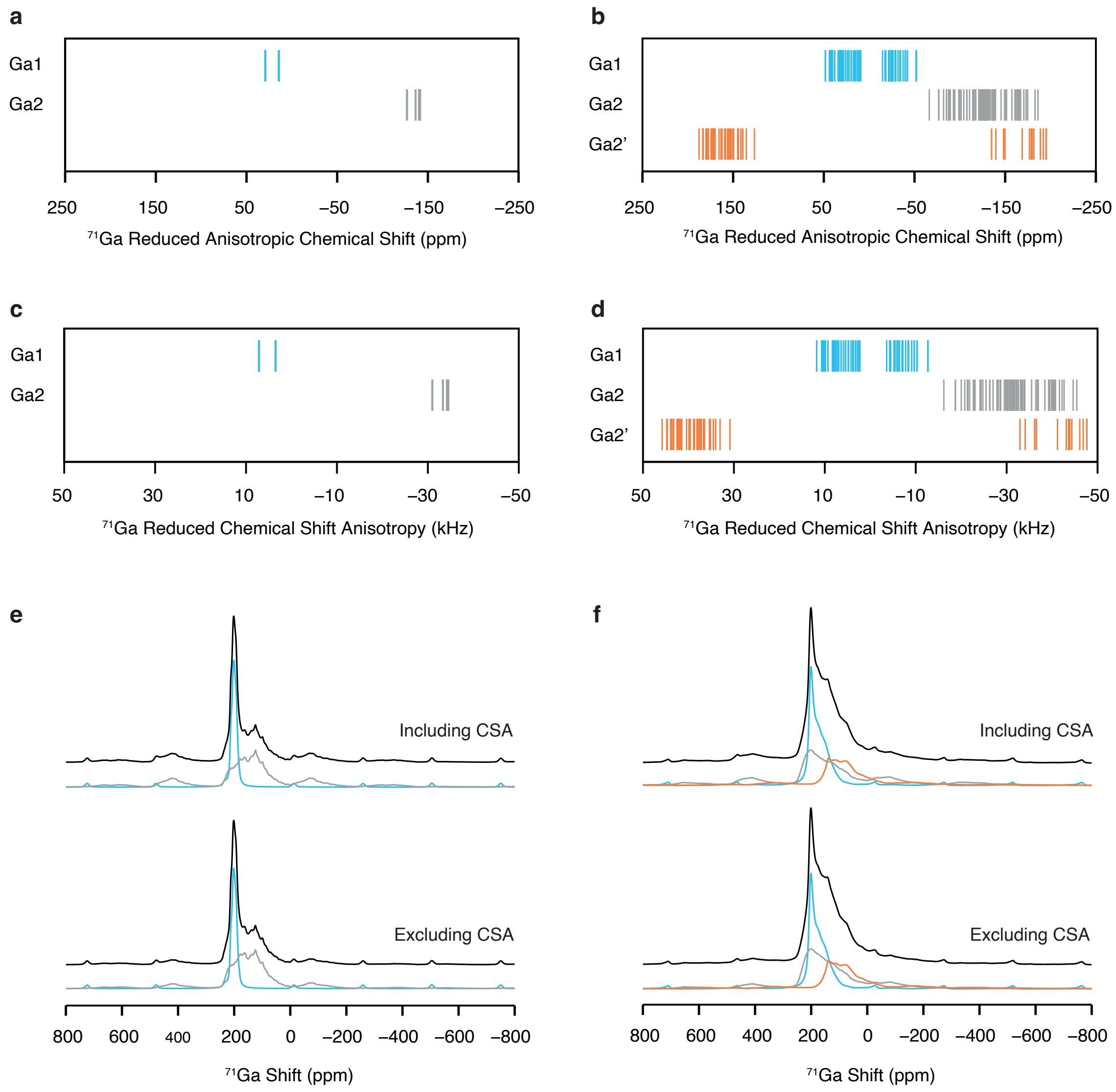

Figure S10. ^{71}Ga reduced chemical shift anisotropy in (**a**, **b**) ppm and in (**c**, **d**) kHz at 18.8 T for (**a**, **c**) $LaSrGa_3O_7$ and (**b**, **d**) $La_{1.5}Sr_{0.5}Ga_3O_{7.25}$ predicted using the GIPAW approach and grouped according to the crystallographically distinct sites Ga1 (blue), Ga2 (gray), Ga2' (orange). ^{71}Ga computationally predicted spectra (black lines) of (**e**) $LaSrGa_3O_7$ and (**f**) $La_{1.5}Sr_{0.5}Ga_3O_{7.25}$ simulated including (top) and excluding (bottom) the chemical shift anisotropy (CSA) at 20 T and under a MAS rate ν_r = 10.0 kHz. The colored lines show the contributions to the total simulated spectra from crystallographically distinct sites. The

contribution of the ^{71}Ga chemical shift anisotropy tensor is negligible and does not significantly affect the ^{71}Ga MAS NMR spectra.

^{71}Ga 3QMAS spectra of $LaSrGa_3O_7$ and $La_{1.54}Sr_{0.46}Ga_3O_{7.27}$ are shown in Figure S11a-b and present one signal assigned to Ga1 which extends to lower frequencies, likely reflecting Czjzek-distributed quadrupolar parameters. Comparison of both spectra reveals that the low frequency contribution to the signal is more pronounced for $La_{1.54}Sr_{0.46}Ga_3O_{7.27}$ than for $LaSrGa_3O_7$, in agreement with the greater spread and overall larger magnitude of the computationally predicted C_Q values for this site. It is evident from the comparison between the ^{71}Ga Hahn echo MAS NMR spectra and the projections of the 2D ^{71}Ga 3QMAS spectra along the f_2 dimension (Figure 12a-b) that in the latter the broad components assigned to Ga2 and Ga2' are absent. This is indicative of inefficient excitation of triple-quantum coherence and subsequent conversion to central transition coherence that result from strong quadrupolar interactions as found computationally for these sites.

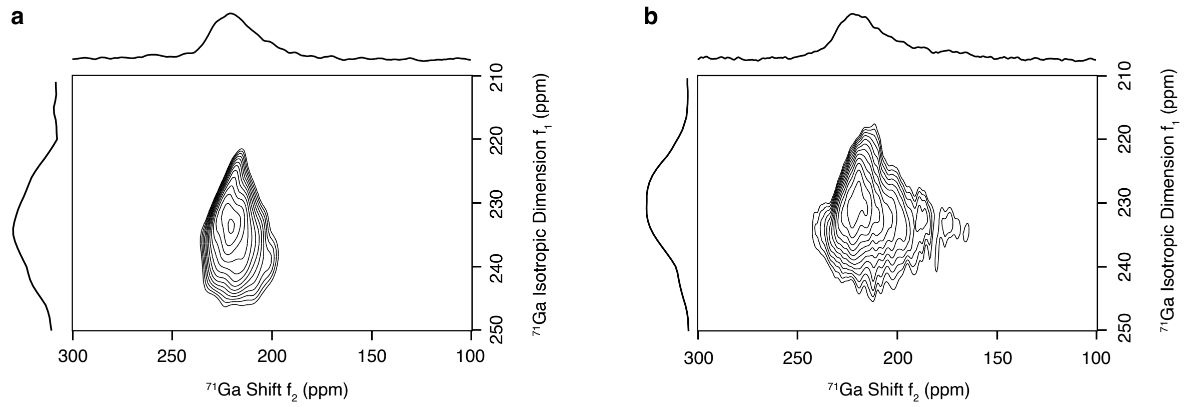

Figure S11. ^{71}Ga 2D 3QMAS[4] spectra of (**a**) $LaSrGa_3O_7$ and (**b**) $La_{1.54}Sr_{0.46}Ga_3O_{7.27}$ recorded at 18.8 T with a MAS rate of ν_r = 60.0 kHz displaying the projections of the 2D 3QMAS spectra along the f_1 and f_2 dimensions.

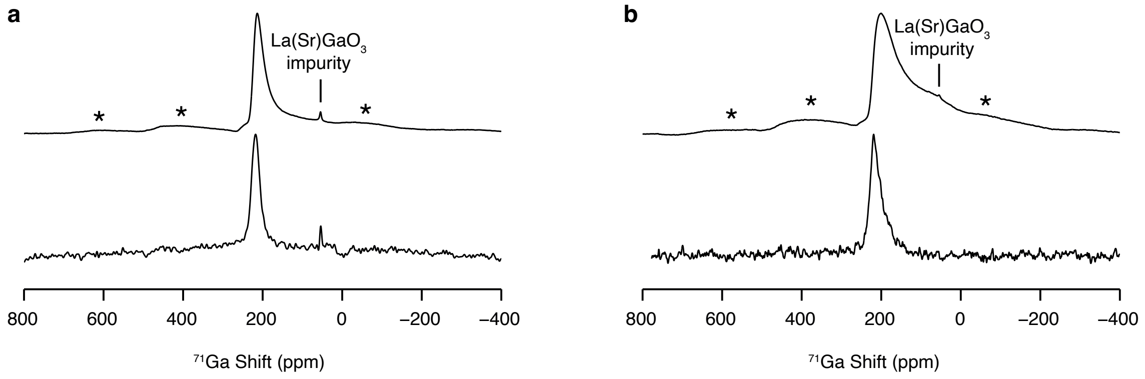

Figure S12. (**a**) $LaSrGa_3O_7$ and (**b**) $La_{1.54}Sr_{0.46}Ga_3O_{7.27}$ ^{71}Ga Hahn echo MAS NMR spectra (top) and projections of the 2D 3QMAS spectra along the f_2 dimensions (bottom) recorded at 18.8 T with a MAS rate of ν_r = 60.0 kHz.

^{139}La and ^{87}Sr NMR spectra were not experimentally acquired in this work owing to the magnitude of the computationally predicted C_Q values (ranging from 48 MHz to 128 MHz for ^{139}La and from 28 MHz to 89 MHz for ^{87}Sr) that would likely lead to broad, significantly overlapped NMR line shapes.

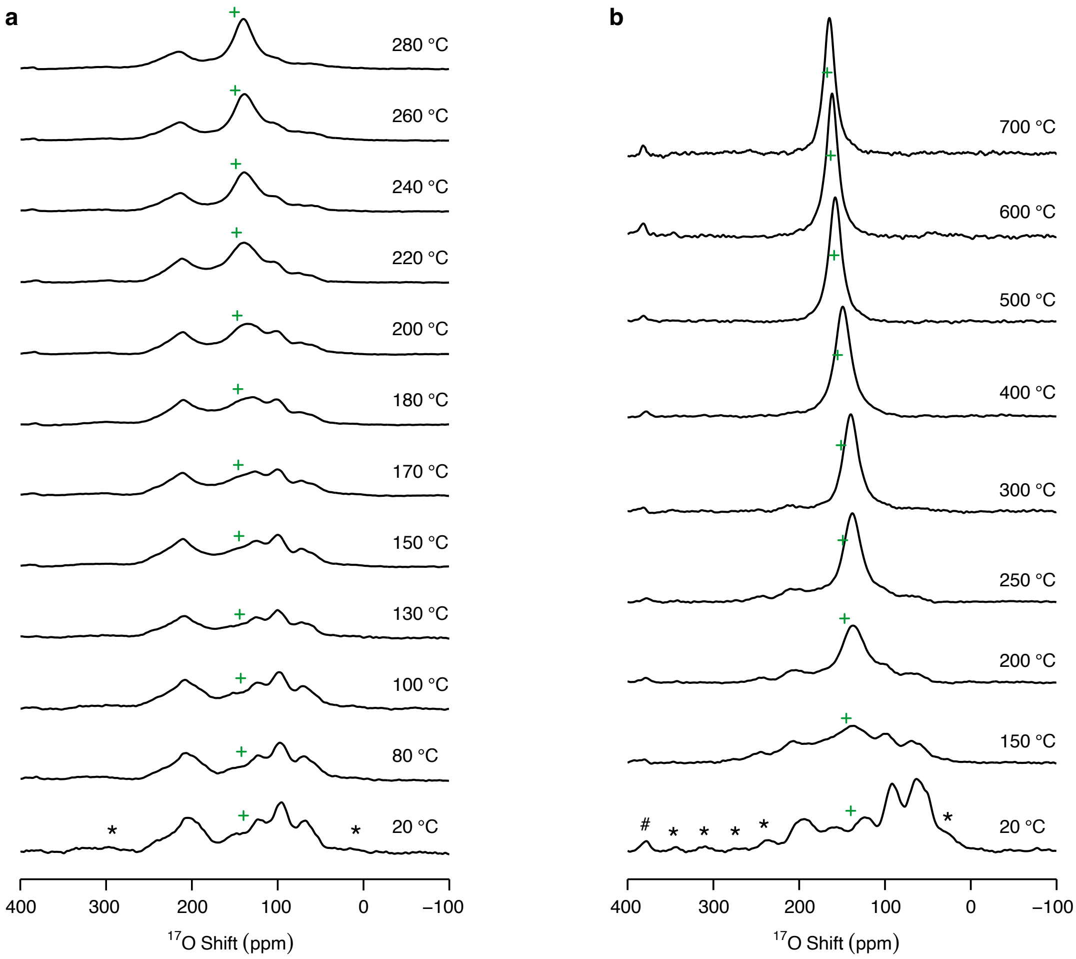

Figure S13. Variable temperature ^{17}O MAS NMR spectra of $La_{1.54}Sr_{0.46}Ga_3{}^{17}O_{7.27}$ recorded at 20 T with (**a**) a 4 mm variable temperature probe with a MAS rate $\nu_r = 10.0$ kHz or (**b**) a 7 mm laser heated probe[7] with a MAS rate $\nu_r = 4.0$ kHz. The green plus symbols (+) denote the weighted average of all resonances as a function of temperature. The hash symbol (#) indicates the ^{17}O signal of the ZrO_2 rotor and the asterisks (*) the spinning sidebands.

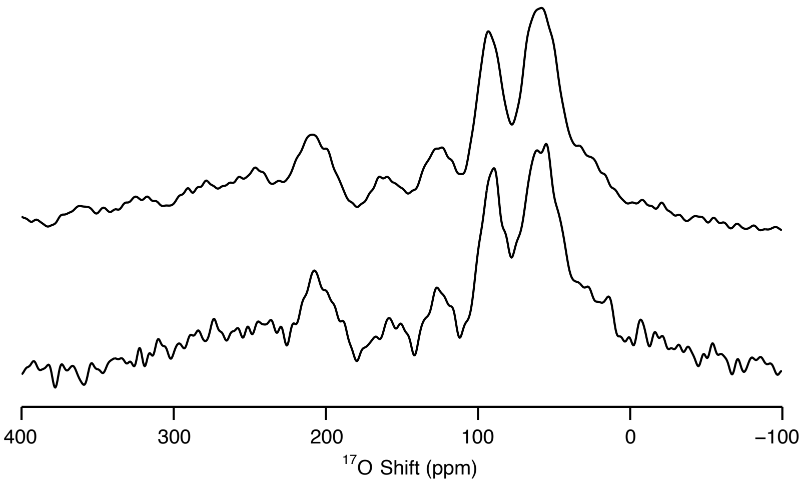

Figure S14. Room temperature ^{17}O MAS NMR spectra of $La_{1.54}Sr_{0.46}Ga_3{}^{17}O_{7.27}$ recorded at 20 T with a 7 mm laser heated probe and a MAS rate ν_r = 4.0 kHz before (bottom, 112 scans) and after (top, 1024 scans) heating up to 700 °C.

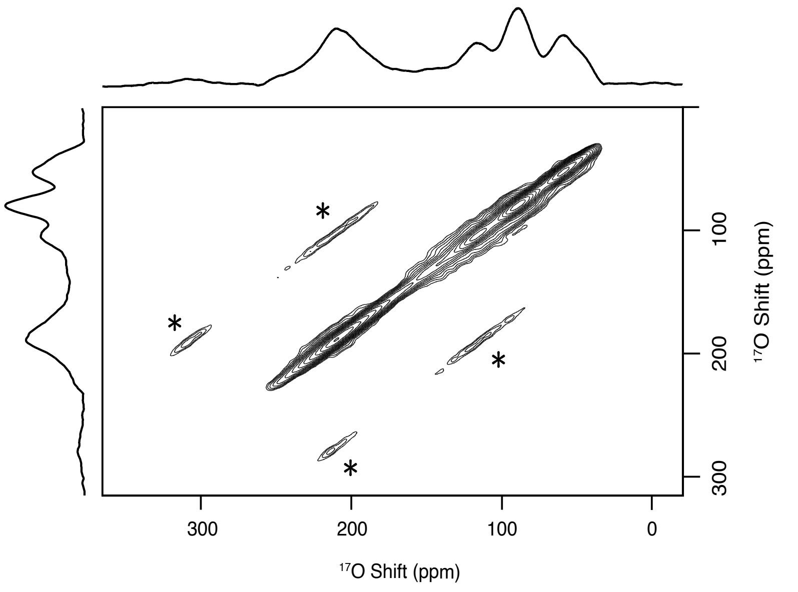

Figure S15. Two-dimensional ^{17}O – ^{17}O EXSY NMR spectrum of $La_{1.54}Sr_{0.46}Ga_3{}^{17}O_{7.27}$ recorded at 20 T at a temperature of 130 °C under a MAS rate of ν_r = 10.0 kHz and with a mixing time of 0 ms. Spinning sidebands are marked with asterisks (*).

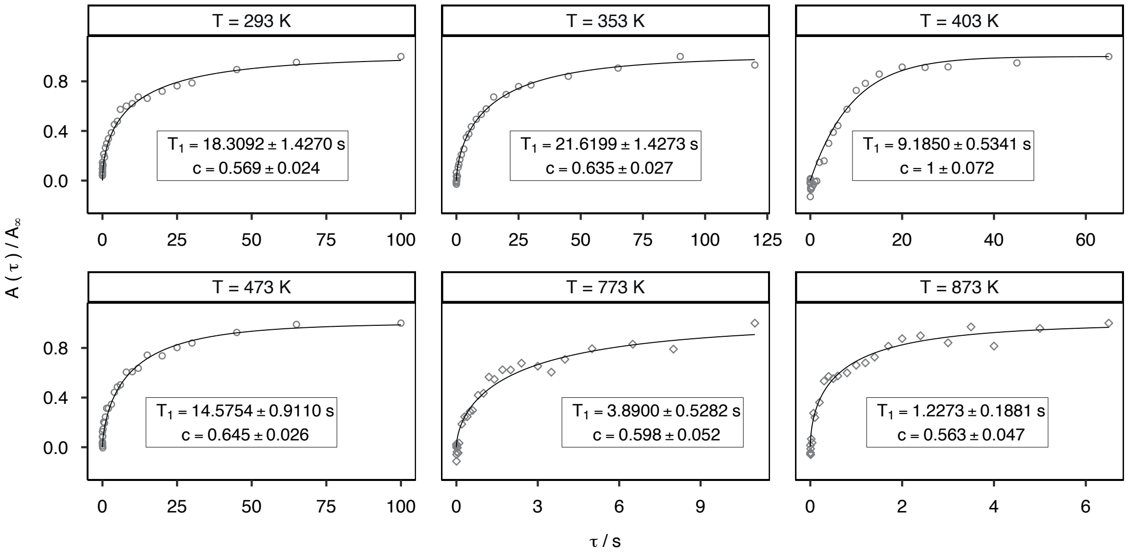

Figure S16. Normalized area $A(\tau)/A(\infty)$ of the $LaSrGa_3{}^{17}O_7$ signals as a function of delay τ obtained from ^{17}O saturation recovery experiments recorded at variable temperature T on a 20 T spectrometer either with the 7 mm laser heated probe under MAS rate ν_r = 4.0 kHz (diamonds) or the 4 mm high temperature probe under MAS rate ν_r = 4.0 kHz (circles) fitted to a stretch exponential function as described in the experimental section. The T_1 and stretch component c values extracted from the fits are reported in the corresponding plots.

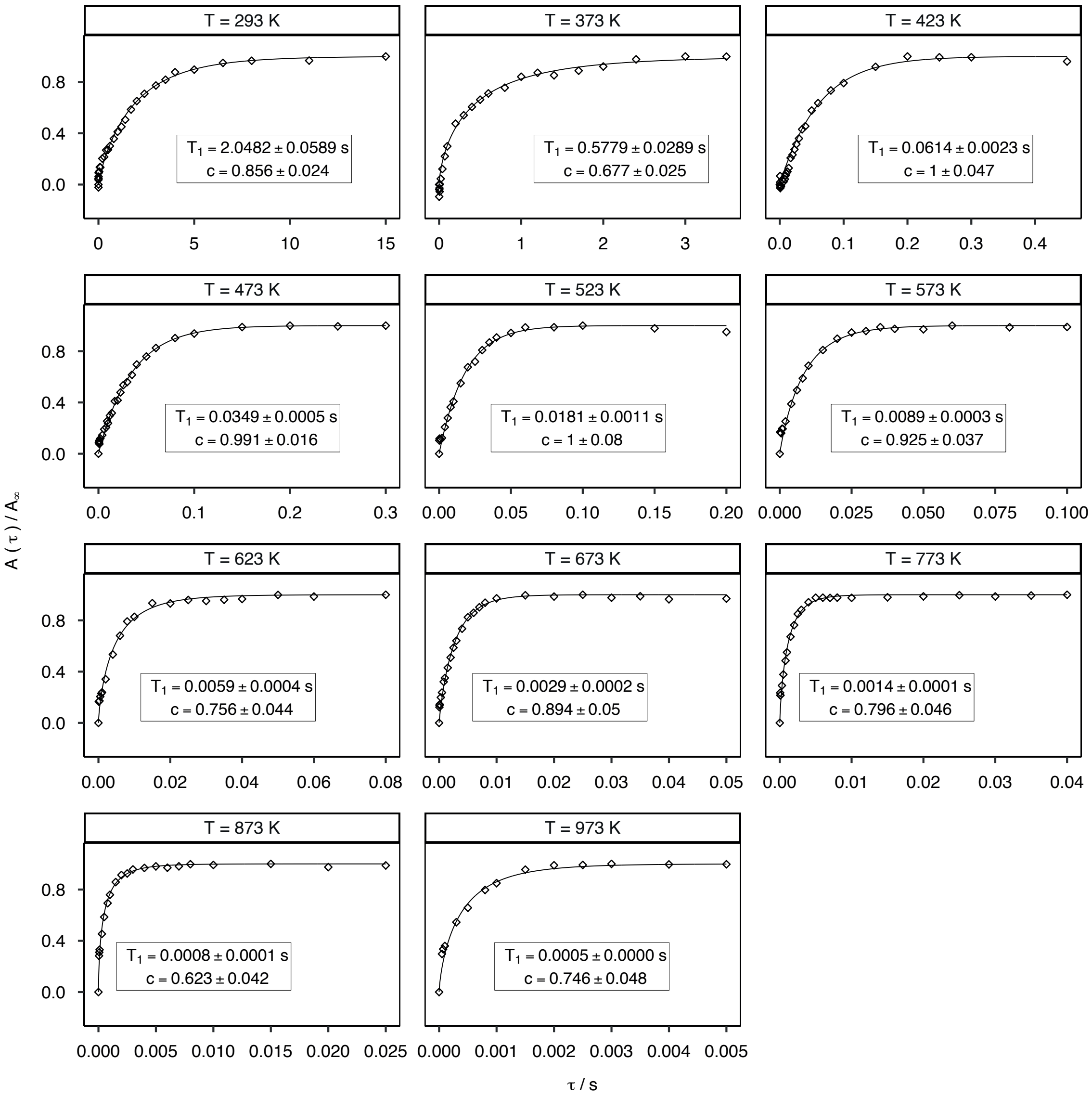

Figure S17. Normalized area $A(\tau)/A(\infty)$ of the $La_{1.54}Sr_{0.46}Ga_3{}^{17}O_{7.27}$ signals as a function of delay τ obtained from ^{17}O saturation recovery experiments recorded with the 7 mm laser heated probe at variable temperature T on a 20 T spectrometer under MAS rate ν_r = 4.0 kHz fitted to a stretch exponential function as described in the experimental section. The T_1 and stretch component c values extracted from the fits are reported in the corresponding plots.

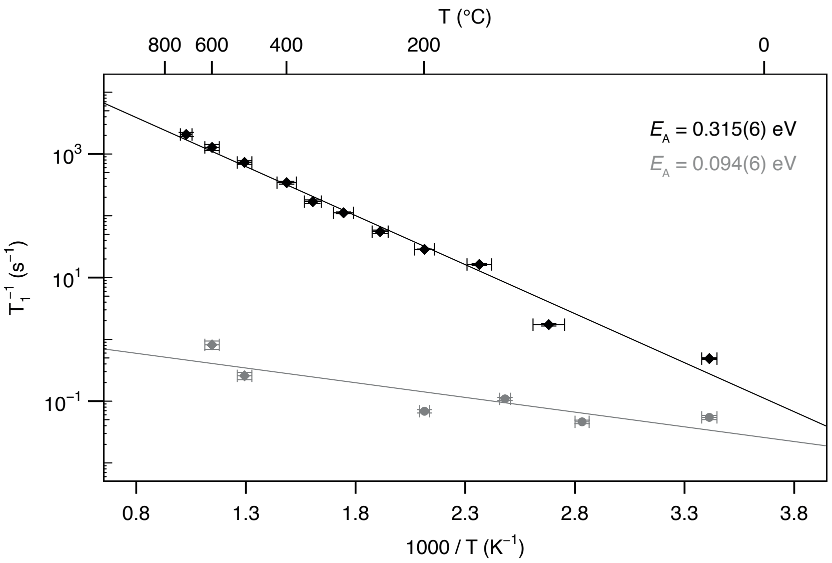

Figure S18. ^{17}O spin-lattice relaxation rates as a function of reciprocal temperature *T* of $La_{1.54}Sr_{0.46}Ga_3{}^{17}O_{7.27}$ (black) and $LaSrGa_3{}^{17}O_7$ (gray) recorded at 20 T either with the 7 mm laser heated MAS probe (diamonds) or the 4 mm high temperature probe (circles), highlighting the corresponding values for the activation energy E_A.

Structural models from DFT calculations and experimental data are accessible from the University of Liverpool Data catalogue: https://doi.org/10.17638/datacat.liverpool.ac.uk/2229.

References.

1. Skakle, J. M. S.; Herd, R., Crystal Chemistry of (RE, A)$_2$M$_3$O$_7$ Compounds (RE=Y, Lanthanide; A=Ba, Sr, Ca; M=Al, Ga). *Powder Diffr.* **1999,** *14* (3), 195-202.
2. Kuang, X.; Green, M. A.; Niu, H.; Zajdel, P.; Dickinson, C.; Claridge, J. B.; Jantsky, L.; Rosseinsky, M. J., Interstitial Oxide Ion Conductivity in the Layered Tetrahedral Network Melilite structure. *Nat. Mater.* **2008,** *7* (6), 498-504.
3. Blanc, F.; Middlemiss, D. S.; Gan, Z.; Grey, C. P., Defects in Doped LaGaO$_3$ Anionic Conductors: Linking NMR Spectral Features, Local Environments, and Defect Thermodynamics. *J. Am. Chem. Soc.* **2011,** *133* (44), 17662-17672.
4. Medek, A.; Harwood, J. S.; Frydman, L., Multiple-Quantum Magic-Angle Spinning NMR: A New Method for the Study of Quadrupolar Nuclei in Solids. *J. Am. Chem. Soc.* **1995,** *117* (51), 12779-12787.
5. Grau-Crespo, R.; Hamad, S.; Catlow, C. R. A.; de Leeuw, N. H., Symmetry-Adapted Configurational Modelling of Fractional Site Occupancy in Solids. *J. Phys. Condens. Matter* **2007,** *19* (25), 256201.
6. Moran, R. F.; McKay, D.; Tornstrom, P. C.; Aziz, A.; Fernandes, A.; Grau-Crespo, R.; Ashbrook, S. E., Ensemble-Based Modeling of the NMR Spectra of Solid Solutions: Cation Disorder in Y$_2$(Sn,Ti)$_2$O$_7$. *J. Am. Chem. Soc.* **2019,** *141* (44), 17838-17846.
7. Ernst, H.; Freude, D.; Mildner, T.; Wolf, I., Laser-Supported High-Temperature MAS NMR for Time-Resolved In Situ Studies of Reaction Steps in Heterogeneous Catalysis. *Solid State Nucl. Magn. Reson.* **1996,** *6* (2), 147-156.
